# Rheology of Cellulose Nanocrystal and Fumed Silica Suspensions: Influence of Ionic Surfactants

**DOI:** 10.3390/nano16110676

**Published:** 2026-05-28

**Authors:** Rajinder Pal, Joshua Richards, Anuva Pal

**Affiliations:** Department of Chemical Engineering, University of Waterloo, Waterloo, ON N2L 3G1, Canada

**Keywords:** cellulose nanocrystals, nanocrystalline cellulose, fumed silica, surfactant, suspension, rheology, viscosity, power law model, non-Newtonian, shear-thinning

## Abstract

Nanomaterials such as cellulose nanocrystals and fumed silica are emerging as excellent thickeners for liquids in a variety of practical applications. Surfactants are often incorporated into the thickening fluids to provide stabilizing components and to control the surface activity of fluids. To develop new thickening materials with desired surface-active properties, it is important to understand the interactions between surfactants and nanoparticles in suspensions. In this work, the interactions between surfactants and nanocrystals/nanoparticles were investigated. Two surfactants, anionic sodium lauryl sulfate-based surfactant (referred to as Stepanol) and cationic hexadecyltrimethylammonium bromide (referred to as HTAB), were studied. Cellulose nanocrystals (referred to as NCC) and fumed-silica nanoparticles (referred to as N20) were used as nanomaterials. The unique feature of this study is that it simultaneously measures rheology, surface activity, and electrical conductivity to determine the influence of ionic surfactants on the behavior and properties of cellulose nanocrystal and fumed silica nanoparticle suspensions. Furthermore, the interactions are observed in the low surfactant concentration range of 0 to 500 ppm. The NCC concentration of NCC–surfactant mixtures was fixed at 1 wt%. Two concentrations of N20 (2 and 5 wt%) were used for N20–surfactant mixtures. The influence of Stepanol was found to be weak whereas HTAB had a strong influence on the rheology of NCC and N20 suspensions. The NCC suspension and surfactant–NCC suspensions were highly non-Newtonian shear-thinning. The N20 suspensions and N20-Stepanol mixtures were nearly Newtonian. The N20-HTAB mixtures were shear-thinning at high HTAB concentrations. The power law model described the rheological behavior of non-Newtonian systems adequately. The consistency and flow behavior indices varied only marginally with the addition of the anionic surfactant Stepanol to NCC and N20 suspensions. With the addition of cationic surfactant HTAB to NCC and N20 suspensions, however, a large increase (20- to 70-fold) in consistency index was observed at high surfactant concentrations. The critical surfactant concentrations where sharp transitions in the rheological properties took place were identified using break points in surface tension and electrical conductivity plots. This study offers valuable insights into tailoring surfactant–nanoparticle systems for practical applications, where precise control of rheological and interfacial properties may be required.

## 1. Introduction and Literature Review

Rheology plays an important role in the formulation and characterization of numerous consumer products [1,2,3,4,5,6]. For example, in the food industry, rheology is essential for developing products with desired textures and consistencies, such as sauces, dressings, and dairy products [4]. It helps in the development of products that have the right mouthfeel and stability during processing and storage [5]. Likewise, in the pharmaceutical industry, rheology plays a vital role in the formulation of drugs, their processing, administration, and controlled release of active ingredients [6]. In the cosmetics industry, the rheological properties of the product influence the application and feel of the products such as lotions, creams, and gels. Control of the rheology of products ensures that the products can spread easily and feel pleasant on the skin. Overall, rheological characterization bridges fundamental science and practical applications [6]. In the case of suspensions of particles, rheology provides valuable insights into how particle interactions, dispersion state, and network formation influence viscosity, yield stress, and flow behavior of suspensions [7]. Such information is vital in designing materials for adhesives, coatings, inks, pharmaceuticals, and construction products, where precise flow control directly impacts processability and final product quality. By understanding the flow and deformation behavior of materials, scientists and engineers can design and optimize products and processes in terms of performance, quality, and user experience [6].

Thus, understanding and controlling the rheology of suspensions of particles—with surfactants as a reinforcing or stabilizing component—is essential for optimizing their performance in practical applications [7,8]. To interpret rheological results accurately, complementary measurements of surface tension and electrical conductivity are also necessary. Surface tension reflects interfacial interactions between suspended particles and the liquid medium, while conductivity reveals the ionic environment and potential electrostatic stabilization. Together, these measurements help explain the rheological behavior in complex suspensions of particles. Furthermore, the dispersed particles tend to agglomerate due to strong hydrogen bonding and van der Waals forces, which compromise dispersion stability and rheological uniformity [9,10,11]. Therefore, studying the influence of surfactants becomes crucial. Surfactants can modify surface charge, reduce attractive forces, and promote better dispersion, resulting in enhanced flow consistency and improved performance of suspension [12,13]. Consequently, it is important to understand how different surfactant types (anionic, cationic, or non-ionic) affect the rheological behavior of suspensions of particles for the tailoring of rheological properties for diverse industrial applications [14,15,16].

In this work, suspensions of nanocrystalline cellulose and fumed silica nanoparticles are investigated. Nanocrystalline cellulose (NCC), also referred to as cellulose nanocrystals (CNC), is an emerging, cost-effective nanomaterial with numerous applications [17,18,19,20,21,22,23,24]. Both the terms NCC and CNC describe the same highly crystalline rod-like nanoparticles derived from cellulose through a process called hydrolysis. In this paper, the abbreviation NCC is used throughout. NCC has recently gained significant attention as a rheology modifier and thickening agent for the matrix phase of emulsions and suspensions, owing to its surface charge and elongated shape with a high aspect ratio [7]. NCC is derived from cellulose. It is typically produced through controlled acid hydrolysis of cellulose fibers [25,26,27,28,29]. The cellulose fibers are treated with acids such as sulfuric acid or hydrochloric acid, which preferentially hydrolyze the amorphous regions of the cellulose, leaving behind the crystalline regions. This process of hydrolysis results in the release of nanocrystals, which are then purified to remove excess acid and other by-products. The nanocrystals produced in this manner have widths typically ranging from 3 to 50 nm and lengths up to several hundred nanometers. The nanocrystals exhibit a highly crystalline structure with glucose units aligned parallel along their longitudinal axis, imparting exceptional mechanical properties such as high tensile strength and stiffness that rival or surpass many synthetic fibers and nanomaterials [17,18]. NCC is also notable for its biodegradability and biocompatibility, making it an environmentally friendly alternative to petroleum-based materials. Derived from renewable resources, it offers sustainable advantages across various industries. The surface chemistry of nanocrystals allows for facile functionalization, enabling attachment of different functional groups, polymers, or nanoparticles to tailor their properties for specific applications. This versatility extends their compatibility with diverse matrices and enhances their utility in fields ranging from biomedical to industrial applications [19,20,21,22]. NCC is widely employed as a reinforcing agent in composite materials, enhancing mechanical performance in automotive, aerospace, and packaging sectors [23,24]. It contributes to the development of lightweight, high-performance materials crucial for advanced technologies. NCC is also used in biomedical applications such as drug delivery systems, tissue engineering scaffolds, and wound-healing materials due to their biocompatibility and ability to facilitate controlled drug release. The unique thickening properties and rheology (shear-thinning non-Newtonian behavior) further broaden their application scope in coatings, films, suspensions, emulsions, and other formulations requiring viscosity control and stability [21,22,23,24].

**Figure 1:**
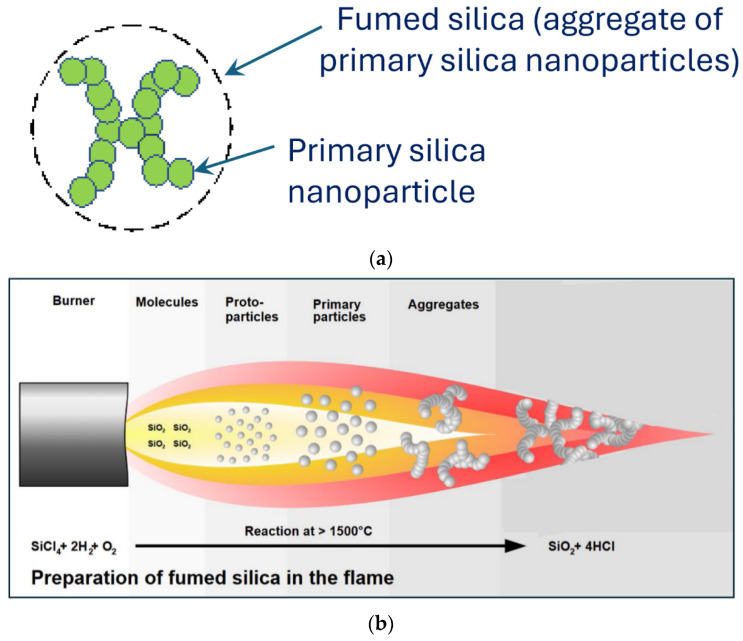
(**a**) Fumed silica microaggregate. (**b**). Generation of fumed silica microaggregates by flame hydrolysis of silicon tetrachloride.

Suspensions of fumed silica nanoparticles were also investigated in this work. Figure 1a shows a single fumed silica nanoparticle. It consists of aggregate primary silica nanoparticles. Fumed silica is a versatile nanomaterial used widely in cosmetics, food, and pharmaceuticals. It has excellent thickening properties. It is produced by flame hydrolysis of silicon tetrachloride (see Figure 1b) [30]. Fused silica nanoparticles are negatively charged in aqueous phase due to ionization of silanol groups (Si-OH) present on their surface.

**Figure 2:**
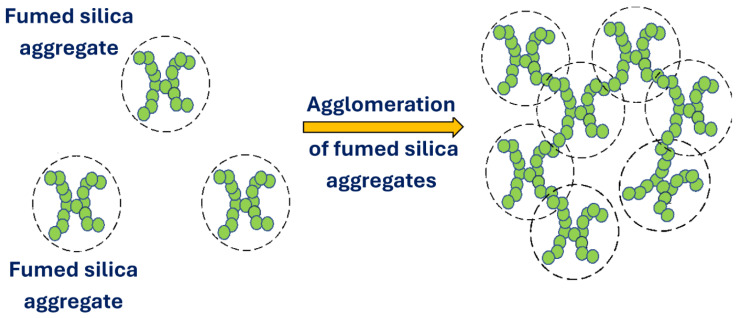
Agglomeration of fumed silica microaggregates.

Note that fumed silica microaggregates usually form large agglomerates in the aqueous phase due to attraction between the fumed silica microaggregates resulting from hydrogen bonding of silanol groups. The large agglomerates of fumed silica microaggregates are illustrated in Figure 2.

The rheological behavior of pure NCC suspensions and pure fumed silica suspensions, without any added surfactant, have been studied extensively [7,8,9,10,11,12,13,14]. For example, Kinra and Pal [7] and others [8,9] investigated the rheology of concentrated NCC suspensions. The NCC concentration varied from 1.03 to 7.41 wt% based on the aqueous phase [7]. The NCC suspensions were shear-thinning and could be described satisfactorily using the power law model. The consistency index increased substantially whereas the flow behavior index decreased sharply with the increase in NCC concentration. A detailed review of stability and rheological properties of fumed and colloidal silica suspensions in aqueous solutions is given by Kawaguchi [10]. The volume fraction of particles was restricted to less than 0.1. The fumed silica suspensions generally exhibit shear-thinning behavior, and the rheology is dependent on the acidic/alkaline conditions of the matrix aqueous phase. Alaee et al. [11] presented a detailed experimental study of shear-thickening behavior and microstructure evolution in concentrated fumed silica suspensions.

The nanocellulose–surfactant interactions are discussed by Tardy et al. [12]. While anionic surfactants can adsorb onto the less charged regions of NCC surfaces via hydrophobic interactions, cationic surfactants lead to the strongest interactions with anionic nanocellulose due to opposite charges. However, the influences of ionic surfactants on surface activity, electrical conductivity, and rheology of suspensions is not discussed to any significant extent.

**Figure 3:**
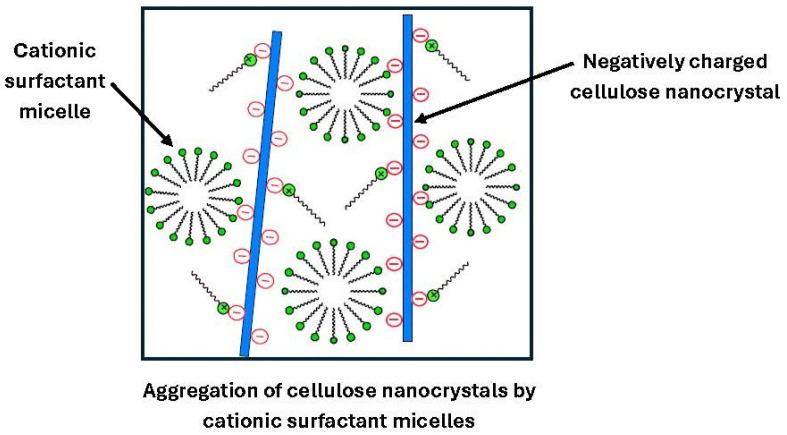
Aggregation of nanocrystals by micelles of cationic surfactant.

Ranjbar and Hatzikiriakos [13] studied the effect of ionic surfactants on the viscoelastic properties of cellulose nanocrystal suspensions. The storage and loss moduli of NCC suspensions increased with the increase in cationic surfactant (cetyltrimethylammonium bromide) concentration. The increases in moduli were explained in terms of aggregation of nanocrystals caused by cationic surfactant micelles as shown schematically in Figure 3.

With the addition of an anionic surfactant (sodium dodecyl sulfate) to the NCC suspension, complicated viscoelastic behavior was observed [13]. The complex modulus exhibited three distinct regions with an increase in surfactant concentration. The complex modulus increases initially with the increases in surfactant concentration, reaches a maximum value at 0.25 mM surfactant concentration, and then it drops by almost an order of magnitude and reaches a minimum value at a surfactant concentration of 2 mM. It rises again with further increase in surfactant concentration. The fluctuation in the complex modulus was explained in terms of competition between hydrophobic and electrostatic interactions between surfactant and nanocrystals.

Gorbacheva and Ilyin [14] studied the effect of the anionic surfactant (sodium dodecyl sulfate) on the rheology of microfibrillated cellulose suspensions and emulsions stabilized by microfibrillated cellulose. With the addition of 5 wt% surfactant to 1 wt% microfibrillar cellulose dispersion in water, the yield stress of cellulose dispersion decreased 10-fold, but there was little change in the high shear rate viscosity. The yield-stress decrease indicated a reduction in interactions between cellulose microfibrils due to screening of hydrophobic parts of cellulose macromolecules by surfactant molecules.

Several papers have been published on the surface modification of colloidal and fumed silica by surfactants [15,16,31]. The silica nanoparticles generally increase the surface activity of anionic surfactants and decrease the surface activity on non-ionic surfactants [15]. While silica nanoparticles are not surface-active alone, they become strongly surface-active by adsorbing cationic surfactant molecules [16]. Furthermore, the cationic surfactants reduce the agglomeration in silica nanoparticles [31]. However, little work has been reported on the influence of surfactants on the rheological properties of fumed silica suspensions.

A good number of papers [32,33,34,35,36,37] have also been published on the modification of the microstructure and rheology of viscoelastic surfactants of worm-like micelles by the incorporation of nanoparticles. The addition of nanoparticles enhances the rheological properties due to interaction of nanoparticles with micellar end caps, thereby building a network structure.

In this work, we explore the interactions between ionic surfactants (cationic as well as anionic) and cellulose nanocrystals and fumed silica nanoparticles. The study is unique in that it simultaneously measures rheology, surface activity, and electrical conductivity to determine the influence of ionic surfactants on the behavior and properties of cellulose nanocrystal and fumed silica nanoparticle suspensions. The interactions are observed in the low surfactant concentration range of 0 to 500 ppm. The surfactant concentration is increased systematically from 0 to 500 ppm in increments of 50 ppm to detect transitions in the behavior and properties of nanosuspensions. The data obtained from rheology, surface activity, and electrical conductivity measurements are considered simultaneously to draw definite conclusions.

## 2. Materials and Methods

### 2.1. Nanocrystalline Cellulose

NCC used in this work was purchased from CelluForce Inc. (Windsor, QC, Canada) in the form of a dry white powder under the trade name of CelluRods 100P. The mean length of the rod-shaped nanocrystals of NCC was 76 nm and the mean width was 3.4 nm, as reported by the company based on atomic force microscopy. The zeta potential of cellulose nanocrystals is generally in the range of −20 to −50 mV [38].

### 2.2. Fumed Silica Nanoparticles

The fumed silica was provided by Wacker Chemie AG (Munchen, Germany). It is synthetic hydrophilic amorphous pyrogenic silica, referred to as N20, produced via flame hydrolysis (see Figure 1b).

### 2.3. Surfactants

Sodium-lauryl-sulfate-based anionic surfactant was supplied as a dry white powder under the trade name of Stepanol WA-100 by Stepan Company, Northfield, IL, USA. It is referred to as Stepanol in the paper. The chemical formula of the surfactant is CH_3_(CH_2_)_10_ CH_2_OSO_3_Na. It consists of 97.59% actives (sodium lauryl sulfate), 0.38% unsulfated material, 0.02% sodium chloride, and 0.56% sodium sulfate.

Hexadecyltrimethylammonium bromide, a cationic surfactant, was supplied as a dry white powder by Sigma-Aldrich (St. Louis, MO, USA). The chemical formula of the surfactant is CH_3_(CH_2_)_15_N(CH_3_)_3_^+^ Br^−^. It is referred to as HTAB in the paper.

### 2.4. Preparation of Nanosuspensions and Surfactant–Nanosuspension Mixtures

The nanosuspensions were prepared at room temperature (22 ± 1 °C) in batches of approximately 1 kg by adding the required amount of additive (NCC or N20 fumed silica) to deionized water. The mixing was achieved using a variable-speed Gifford-Wood homogenizer (Model 1-L). The mixing was carried out for about 1 h at appropriate speed to ensure complete dispersion of nanocrystals/nanoparticles. For NCC suspensions, the concentration of NCC was fixed at 1 wt%. For fumed silica suspensions, the concentrations of N20 were fixed at 2 wt% and 5 wt%. Thus, NCC suspensions were investigated at a single concentration whereas the N20 suspensions were investigated at two concentrations.

The surfactant–nanosuspension mixtures were prepared at room temperature by adding the known amounts of surfactant to the nanosuspension and carrying out the mixing using the homogenizer at a gentle speed for about 1 h. The surfactant–nanosuspension mixtures were prepared in surfactant concentration increments of 50 ppm by adding more surfactant to an existing surfactant–nanosuspension mixture and carrying out the mixing. The surfactant concentration varied in the range of 0–500 ppm. Care was taken not to entrap any air in the dispersion during the homogenization process. The compositions of the nanosuspensions investigated are summarized in Table 1.

The nanosuspension and surfactant–nanosuspension mixtures were cooled to room temperature before doing any measurements.

### 2.5. Measurement of Steady Shear Rheology of Nanosuspensions and Surfactant–Nanosuspension-Mixtures

A Fann 35A/SR 12 coaxial cylinder viscometer (Fann Instrument Company, Houston, TX, USA) was used to do the rheological measurements. The outer cylinder (rotor) of the viscometer rotated while the inner cylinder (bob) was kept stationary. The inner cylinder had a radius of 1.7245 cm and the outer cylinder had a radius of 1.8415 cm resulting in a shear gap of a width of 0.117 cm. The length of the bob was 3.8 cm. The rotational speed could be varied from 0.9 to 600 rpm corresponding to a shear rate range of approximately 1.53–1021.38 s^−1^. All measurements of viscosities were carried out at room temperature (≈22 °C).

The data for both NCC and silica suspensions were collected in an increasing shear rate mode. Starting from a low shear rate of 1.53 s^−1^, the shear rate was increased progressively to the highest value of 1021.38 s^−1^. At each shear rate, the steady torque reading was recorded. It took a few seconds for the reading to stabilize at any given shear rate. No hysteresis effect was observed.

Calibration of the viscometer was done using viscosity standards of known viscosities supplied by Koehler Instrument Co, New York, NY, USA. The viscosities of the standards used were as follows at 20 °C: S6 (8.551 mPa.s), S60 (134 mPa.s), N350 (992.1 mPa.s), and N1000 (3949 mPa.s). For each standard, the variation in viscosity with temperature was also specified.

### 2.6. Measurement of Surface Tension of Nanosuspension and Surfactant–Nanosuspension Mixtures

**Figure 4:**
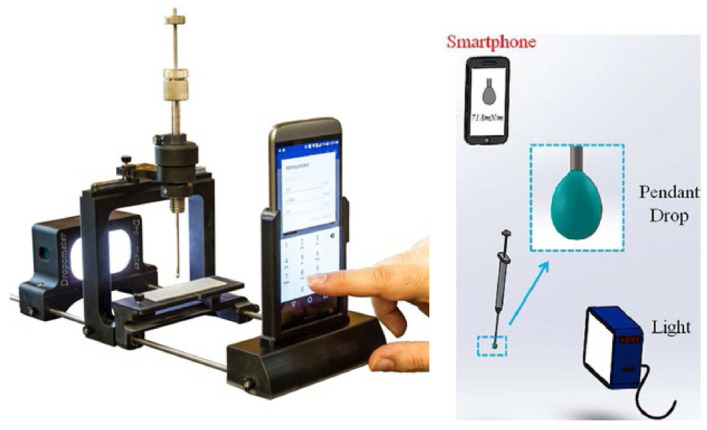
Smartphone-based pendant drop tensiometer.

The surface tension measurement of nanosuspension and surfactant–nanosuspension mixtures was carried out using the pendant drop method with a smartphone-based pendant drop tensiometer manufactured by Droplet Lab, Markham, ON, Canada. A pendant droplet of an aqueous phase (nanosuspension or surfactant–nanosuspension mixture) was generated at the tip of a stainless-steel needle (1.8 mm diameter) connected to a 500 µL Hamilton^®^ gastight syringe (Model 1750 TPLT, Hamilton Company, Reno, NV, USA). The droplet was dispensed using a screw-driven plunger for precise control of flow rate and drop formation. The pendant droplet thus formed was imaged at a high resolution using a smartphone camera and analyzed using specialized software. From the drop shape analysis, the software was able to calculate the surface tension of each solution numerically by fitting the droplet profile with the Young–Laplace equation [39]. The measurement for each fluid was performed at least 13 times to ensure precision and reproducibility, and the average value was calculated. All measurements were done at room temperature. Figure 4 shows the smartphone-based pendant drop tensiometer.

As an example, the measured surface tension for 2 wt% N20 containing 400 ppm Stepanol is as follows: 50.47, 54.82, 50.4, 50.56, 50.7, 50.67, 50.97, 50.26, 51.07, 50.64, 50.86, 50.51, and 50.72 mN/m. The mean value of surface tension is 50.973 mN/m and the standard deviation is 1.177.

### 2.7. Measurement of Electrical Conductivity of Nanosuspension and Surfactant–Nanosuspension Mixtures

The electrical conductivity of nanosuspension and surfactant–nanosuspension mixtures was measured at room temperature using a Thermo Orion 3 Star conductivity meter (Thermo Fischer Scientific Inc., Beverly, MA, USA).

A Thermo Scientific 013005MD probe with a cell constant of 0.475 cm^−1^ was used. The calibration standard used was 692 ppm NaCl solution with a conductivity of 1413 µS/cm.

### 2.8. Size Distribution of Cellulose Nanocrystals (NCC) and Fumed Silica

To determine the size distribution of cellulose nanocrystals (NCC) and fumed silica (N20), the dynamic light scattering (DLS) technique was used. The measurements were carried out using a Zetasizer Nano ZS90 instrument manufactured by Malvern Instruments Ltd. (Malvern, UK). For data acquisition and analysis, Zetasizer 6.20 software was used. The dilute suspensions of NCC and fumed silica in water were tested in ZEN0112 low-volume disposable cuvettes and analyzed at a standard temperature of 25 °C. A 120 s period of equilibration was observed prior to analysis to ensure optimal sample stability.

In the DLS method, the hydrodynamic diameter of the particles is determined from the measurement of the translational diffusion coefficient of particles using dynamic light scattering (DLS). The Stokes–Einstein equation, given below, is used to estimate the hydrodynamic diameter from the measurement of the diffusion coefficient:

(1) $$D=\frac{k_{B}T}{3\pi \eta d_{H}}$$

where D is the diffusion coefficient, $k_{B}$ is the Boltzmann constant, T is temperature, η is the viscosity of the suspending medium (dispersant), and $d_{H}$ is the hydrodynamic diameter. Note that this equation is valid only for spherical particles in the continuum regime. As NCC particles are rod-shaped, the equation should include a shape factor. Thus, the reported “hydrodynamic diameter” is an equivalent spherical diameter, not the actual particle dimension.

## 3. Suspensions of Cellulose Nanocrystals (NCC)

### 3.1. Size Distribution of Cellulose Nanocrystals

**Figure 5:**
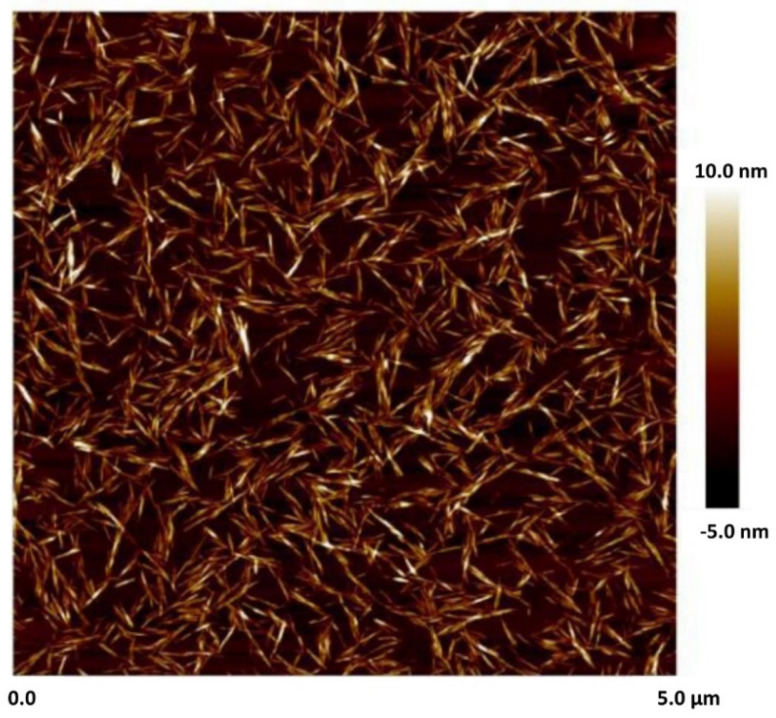
AFM image of nanocrystalline cellulose.

Figure 5 shows the AFM (atomic force microscopy) image of cellulose nanocrystals [7]. Clearly, the nanocrystals are rod-shaped. Note that the image is from the literature for illustrative purposes.

**Figure 6:**
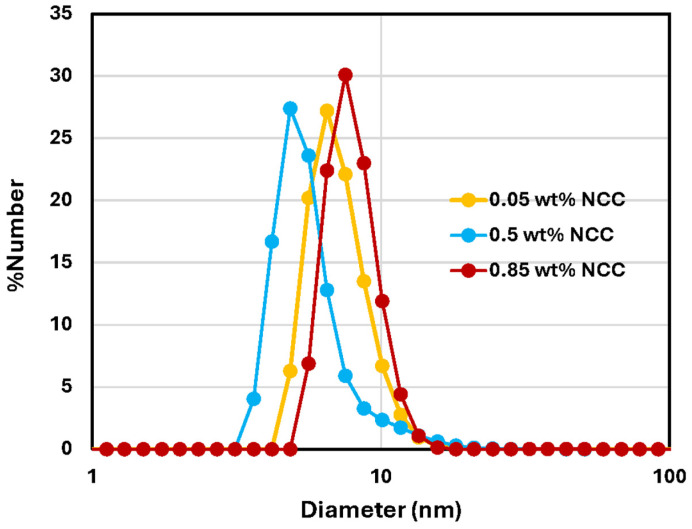
Size distribution of cellulose nanocrystals at NCC concentrations of 0.05, 0.5, and 0.85 wt%.

Figure 6 shows the DLS data for NCC suspension at NCC concentrations of 0.05, 0.5, and 0.85 wt%. The variations in DLS data are likely due to minor aggregation of particles. The average hydrodynamic diameter of NCC crystals is approximately 7 nm. Note that the actual dimensions of the rod-shaped nanocrystals may be quite different from the equivalent hydrodynamic diameter obtained through DLS measurements.

### 3.2. Rheology of Suspensions of Cellulose Nanocrystals

**Figure 7:**
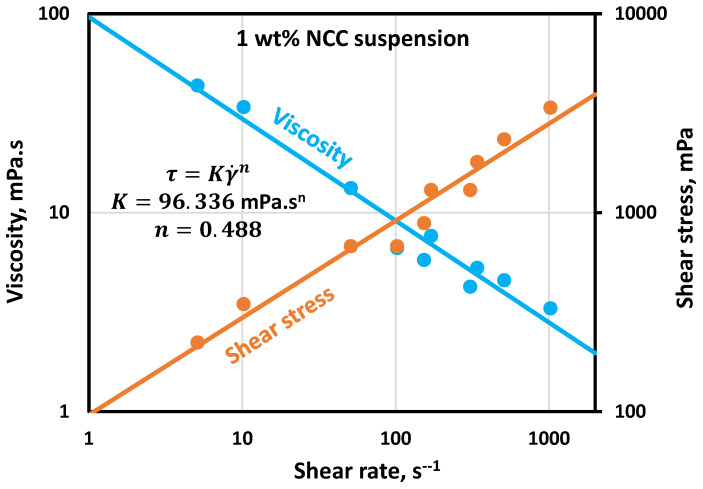
Rheological behavior of 1 wt% NCC suspension.

Figure 7 shows the rheological behavior of 1 wt% NCC suspension. Viscosity decreases with the increase in shear rate indicating the NCC dispersion in non-Newtonian pseudoplastic or shear-thinning. Both viscosity-versus-shear-rate and shear-stress-versus-shear-rate plots are linear on a log-log scale indicating that the NCC suspension follows a power law model [7]:

(2) $$\tau =K{\dot{\gamma }}^{n}$$

(3) $$\eta =\tau /\dot{\gamma }=K{\dot{\gamma }}^{n-1}$$

where τ is shear stress, $\dot{\gamma }$ is shear rate, K is the consistency index, and n is the flow behavior index. K is a measure of consistency of a fluid and n is a measure of flow behavior (Newtonian versus non-Newtonian) of a fluid. Newtonian fluids have n=1 whereas non-Newtonian fluids have an n that is different from unity. For pseudoplastic shear-thinning fluids, n<1, and for dilatant shear-thickening fluids, n>1. It should be noted that the power law model (Equations (1) and (2)) could be recast in the logarithmic form as follows:

(4) $$ln\tau =lnK+nln\dot{\gamma }$$

(5) $$ln\eta =lnK+\left(n-1\right)ln\dot{\gamma }$$

Consequently, the power law fluids exhibit a linear relationship of viscosity-versus-shear-rate and shear-stress-versus-shear-rate on a log-log scale. The slope of the shear stress versus shear rate plot, that is, n, is positive, and the slope of viscosity versus the shear rate plot, that is, $\left(n-1\right)$, is negative.

For the 1 wt% NCC suspension under consideration, n=0.488 and $K=96.3\;mPa.s^{n}$.

### 3.3. Influence of Surfactants on the Rheology of NCC Suspensions

**Figure 8:**
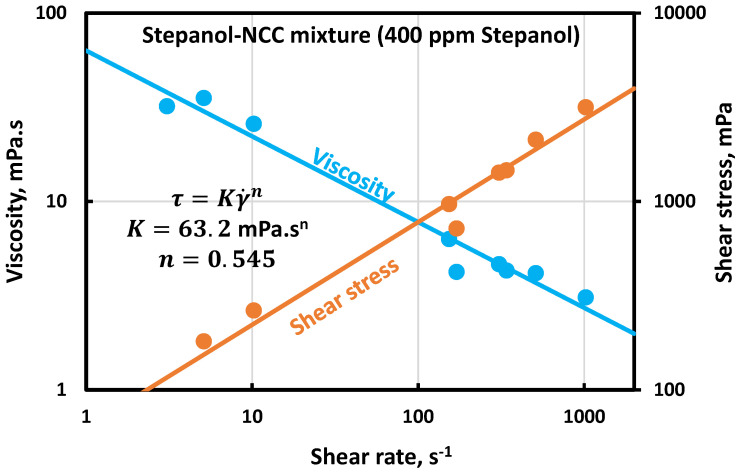
Rheological behavior of Stepanol–NCC mixture containing 400 ppm Stepanol.

**Figure 9:**
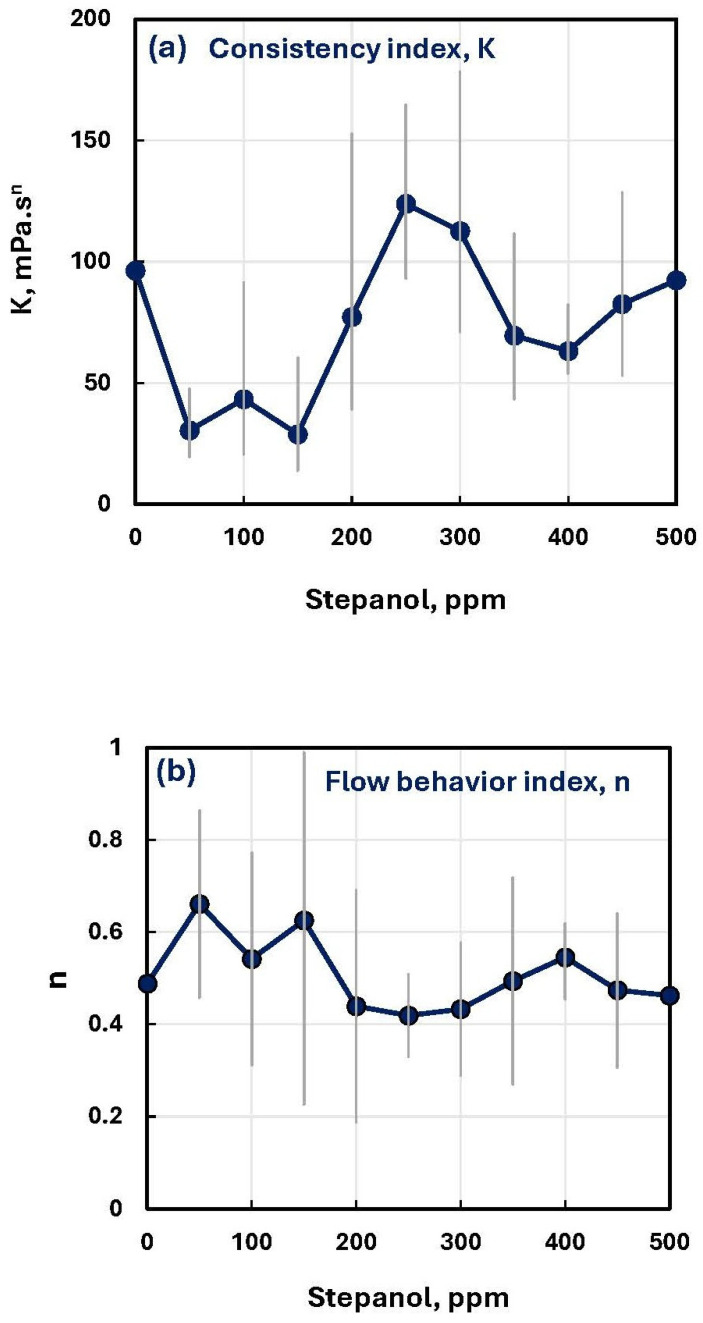
Variations in consistency and flow behavior indices of Stepanol–NCC mixtures with Stepanol concentration. (**a**) Consistency index. (**b**) Flow behavior index.

Figure 8 shows the typical rheological behavior of a Stepanol–NCC mixture. With the addition of the anionic surfactant Stepanol to NCC suspension, the mixtures remain non-Newtonian shear-thinning, that is, the viscosity decreases with the increase in shear rate. Furthermore, the mixtures follow the power law behavior as illustrated in Figure 8. However, a significant amount of scatter in data was observed. The power law parameters, consistency index K and flow behavior index n, for the Stepanol–NCC mixtures, are summarized in Table 2 and plotted in Figure 9. The error bars clearly show large uncertainty in the power law parameters. The mean values of the power law parameters (K and n) fluctuate with the addition of Stepanol to NCC suspension with no clear trend. The variations in K and n are within experimental errors as indicated by the error bars in Figure 9.

**Figure 10:**
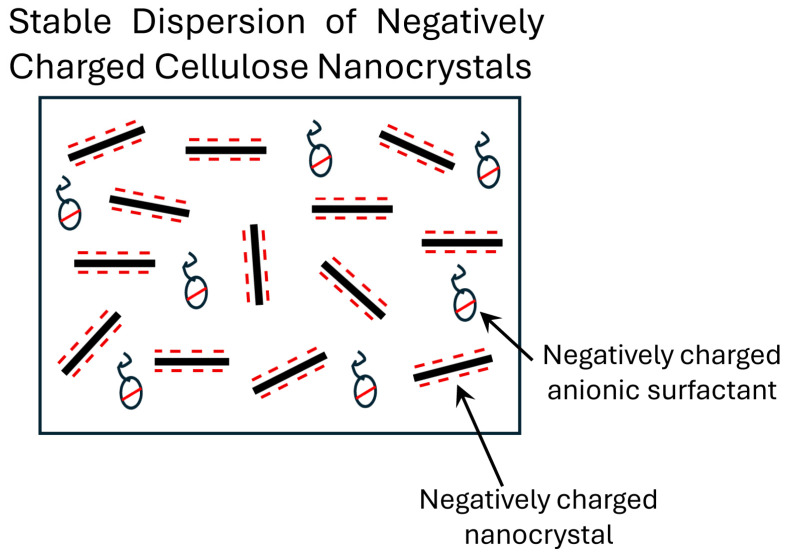
Stable suspension of anionic surfactant and cellulose nanocrystals.

Thus, it can be concluded that the interaction between Stepanol and NCC is weak. The addition of Stepanol to NCC has a marginal effect on rheological properties. As both Stepanol and NCC possess the same electric charge (negative), they repel each other, resulting in a stable suspension as shown schematically in Figure 10.

**Figure 11:**
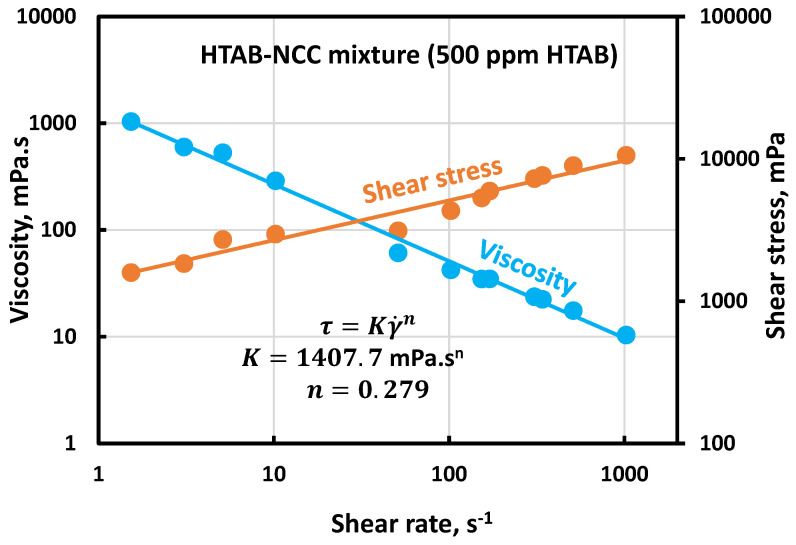
Rheological behavior of HTAB-NCC mixture containing 500 ppm HTAB.

**Figure 12:**
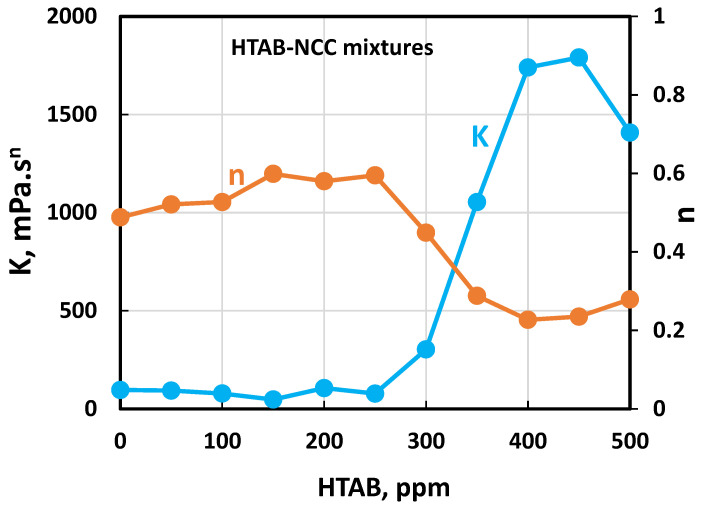
Variations in consistency and flow behavior indices of HTAB-NCC mixtures with HTAB concentration.

**Figure 13:**
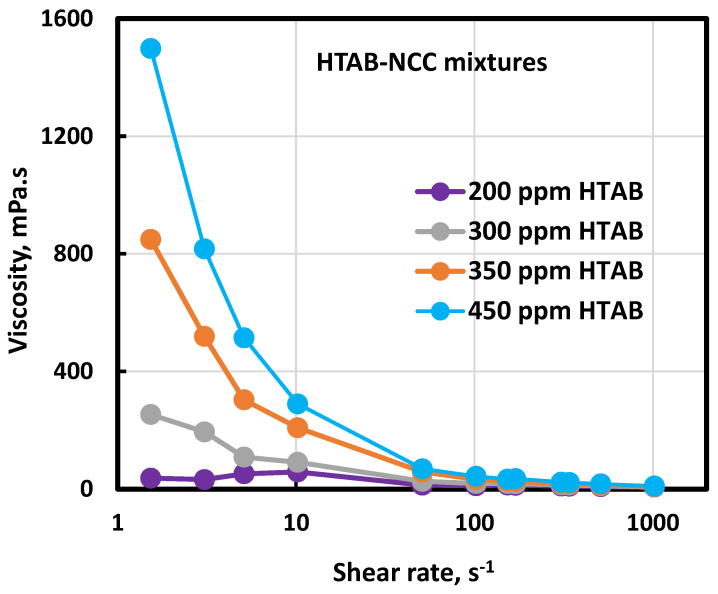
Viscosity versus shear rate plots of HTAB-NCC mixtures at different HTAB concentrations.

Figure 11 shows the typical rheological behavior of an HTAB-NCC mixture. Although the HTAB-NCC mixture remains highly non-Newtonian (shear-thinning) and obeys the power law model with the addition of a cationic surfactant to NCC suspension, the rheological properties are now strongly influenced by the addition of the surfactant to NCC. For example, the consistency index of the HTAB-NCC mixture shoots up to 1790.8 mPa.s^n^ at 450 ppm HTAB in comparison to K= 96.336 mPa.s^n^ for NCC suspension without any surfactant. Furthermore, the degree of shear-thinning of the mixture also increases as indicated by a decrease in flow behavior index from n=0.488 at 0 ppm HTAB to n=0.235 at 450 ppm HTAB. Table 3 summarizes the power law model parameters (K and n) for all HTAB-NCC mixtures. As shown in Figure 12, the consistency index K remains nearly constant up to a surfactant HTAB concentration of about 300 ppm and then shoots up with further increase in HTAB concentration. The flow behavior index n increases slightly up to a HTAB concentration of 300 ppm and then drops significantly with further increase in HTAB concentration, making the mixture more shear-thinning. The viscosity versus shear rate plots of HTAB-NCC mixtures are shown in Figure 13 for different HTAB concentrations. As expected, the viscosities increase sharply with the increase in HTAB concentration, especially at low shear rates.

**Figure 14:**
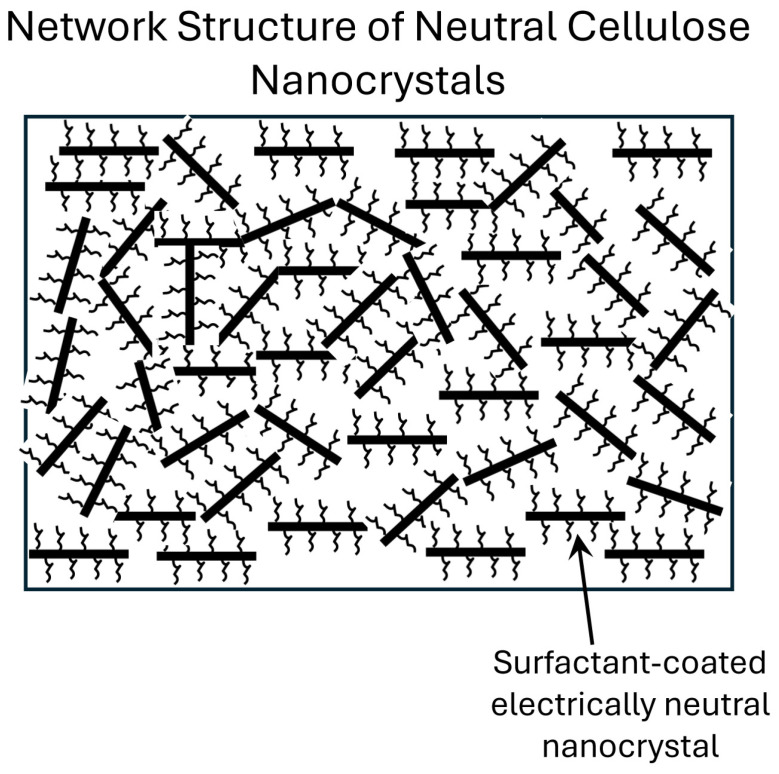
Aggregation/flocculation of charge-neutralized cellulose nanocrystals in the presence of cationic surfactant.

**Figure 15:**
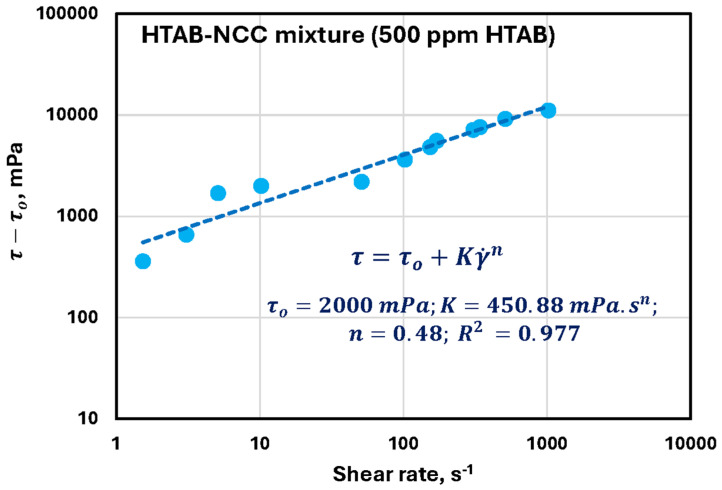
Interpretation of the rheological data of HTAB-NCC mixture (500 ppm HTAB) in terms of the Herschel–Bulkley model.

The sharp increase in consistency or viscosity of HTAB-NCC mixtures with the addition of HTAB above 300 ppm is indicative of the formation of a three-dimensional network structure of cellulose nanocrystals under the influence of cationic surfactant HTAB. As cellulose nanocrystals (negatively charged) and HTAB (positively charged) possess opposite charges, the surfactant molecules neutralize the charge of nanocrystals and become attached to the surface of the nanocrystals. In the absence of any electric repulsion between the cellulose nanocrystals, they favor the formation of a network structure in liquids resulting in gel-like behavior. The gel-like structure in dispersion of charge-neutralized cellulose nanocrystals is depicted schematically in Figure 14. However, this mechanism of the formation of a three-dimensional network structure of cellulose nanocrystals is speculative. Other possible mechanisms of microstructure formation are micelle adsorption on nanocrystals (see Figure 3), bridging of nanocrystals, and depletion flocculation of nanocrystals. Further studies are required to pinpoint the exact mechanism of microstructure formation. Interestingly, the rheological data of HTAB-NCC mixtures at high HTAB concentrations (400 to 500 ppm) could be interpreted in terms of the Herschel–Bulkley model indicating that they possess a yield stress, further supporting the formation of a sample-spanning microstructure. The Herschel–Bulkley model is given as:

(6) $$\tau =\tau_{o}+K{\dot{\gamma }}^{n}$$

where $\tau_{o}$ is the yield stress. As an example, Figure 15 shows the interpretation of the rheological data of an HTAB-NCC mixture containing 500 ppm HTAB in terms of the Herschel–Bulkley model. As can be seen, the data can be fitted to the Herschel–Bulkley model adequately using the yield stress $\tau_{o}$ of 2 Pa.

**Figure 16:**
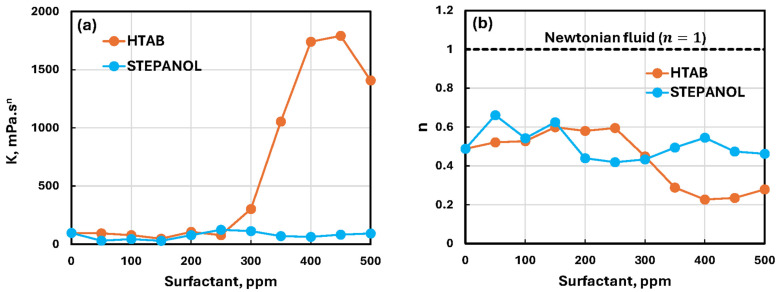
Comparison of the rheological properties of Stepanol–NCC and HTAB-NCC mixtures. (**a**) Consistency index K; (**b**) flow behavior index n.

Figure 16 compares the rheological behaviors of Stepanol–NCC and HTAB-NCC mixtures. There is relatively negligible change in the rheological properties (K and n) of Stepanol–NCC mixtures with the increase in Stepanol concentration. The rheological properties of HTAB-NCC mixtures are also nearly unchanged with the initial increase in surfactant concentration. However, starting at 300 ppm HTAB, the consistency index shoots up and the flow behavior index decreases significantly with further increase in HTAB concentration. The increase in consistency index and the corresponding decrease in flow behavior index is likely to be due to the formation of interconnected microstructure of nanocrystals. Note that all fluids (NCC suspension, surfactant–NC mixtures) are highly non-Newtonian shear-thinning as shown in Figure 16b. The range of flow behavior index is: 0.23≤n≤0.6. Thus, n is way below the Newtonian fluid value of unity.

### 3.4. Influence of Surfactants on the Electrical Conductivity and Surface Tension of NCC Suspensions

**Figure 17:**
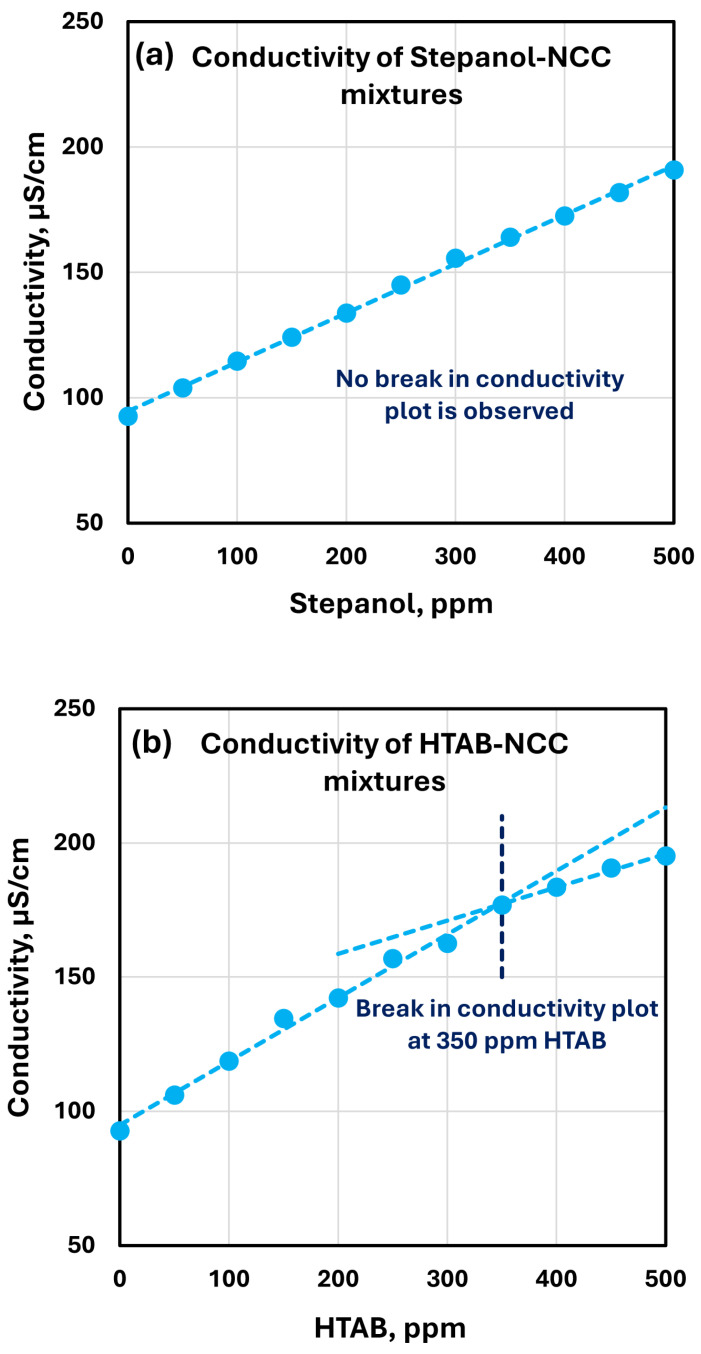
Electrical conductivity variation in surfactant–NCC mixtures with the increase in surfactant concentration. (**a**) Conductivity of Stepanol–NCC mixtures; (**b**) conductivity of HTAB-NCC mixtures.

**Figure 18:**
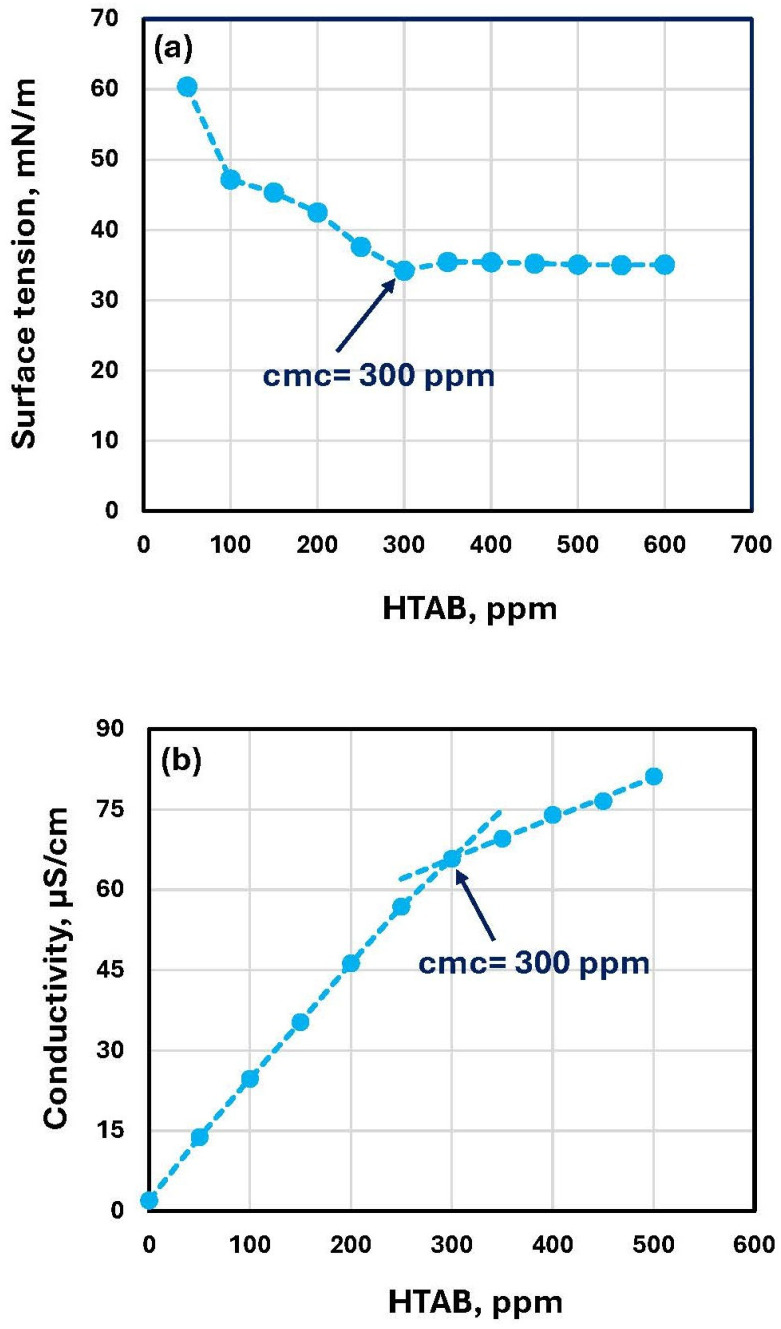
Surface tension and electrical conductivity of pure HTAB solutions. (**a**) Surface tension. (**b**) Electrical conductivity.

The electrical conductivity plots of Stepanol–NCC and HTAB-NCC mixtures are shown in Figure 17. The conductivity of the Stepanol–NCC mixture increases linearly with the increase in Stepanol concentration without any break or change in slope (see Figure 17a). This is consistent with the variation in rheological properties of the Stepanol–NCC mixture with the increase in Stepanol concentration. No clear trend was observed in the changes in rheological properties (see Figure 9). However, the conductivity plot of HTAB-NCC mixtures show a different behavior (see Figure 17b). The conductivity of the HTAB-NCC mixture increases linearly up to about 350 ppm HTAB. At 350 ppm HTAB, a change in the slope of the conductivity plot occurs. At HTAB concentrations higher than 350 ppm, the conductivity increases slowly with the increase in HTAB concentration. This is consistent with the rheological data which exhibited a large change around a HTAB concentration of 300 ppm. At HTAB concentrations larger than 300 ppm, some of the surfactant molecules migrate to the surface of nanocrystals resulting in charge neutralization and hence we observe a slower increase in conductivity with the increase in HTAB concentration. It should be noted that the cmc (critical micelle concentration) of pure HTAB solutions is reported to be 0.91 mM [40]. This corresponds to 332 ppm. The surface tension and electrical conductivity data obtained in our lab give a cmc of 300 ppm (see Figure 18). Thus, the changes in the rheological properties of HTAB-NCC mixtures occur when the surfactant concentration exceeds the cmc of a pure surfactant.

**Figure 19:**
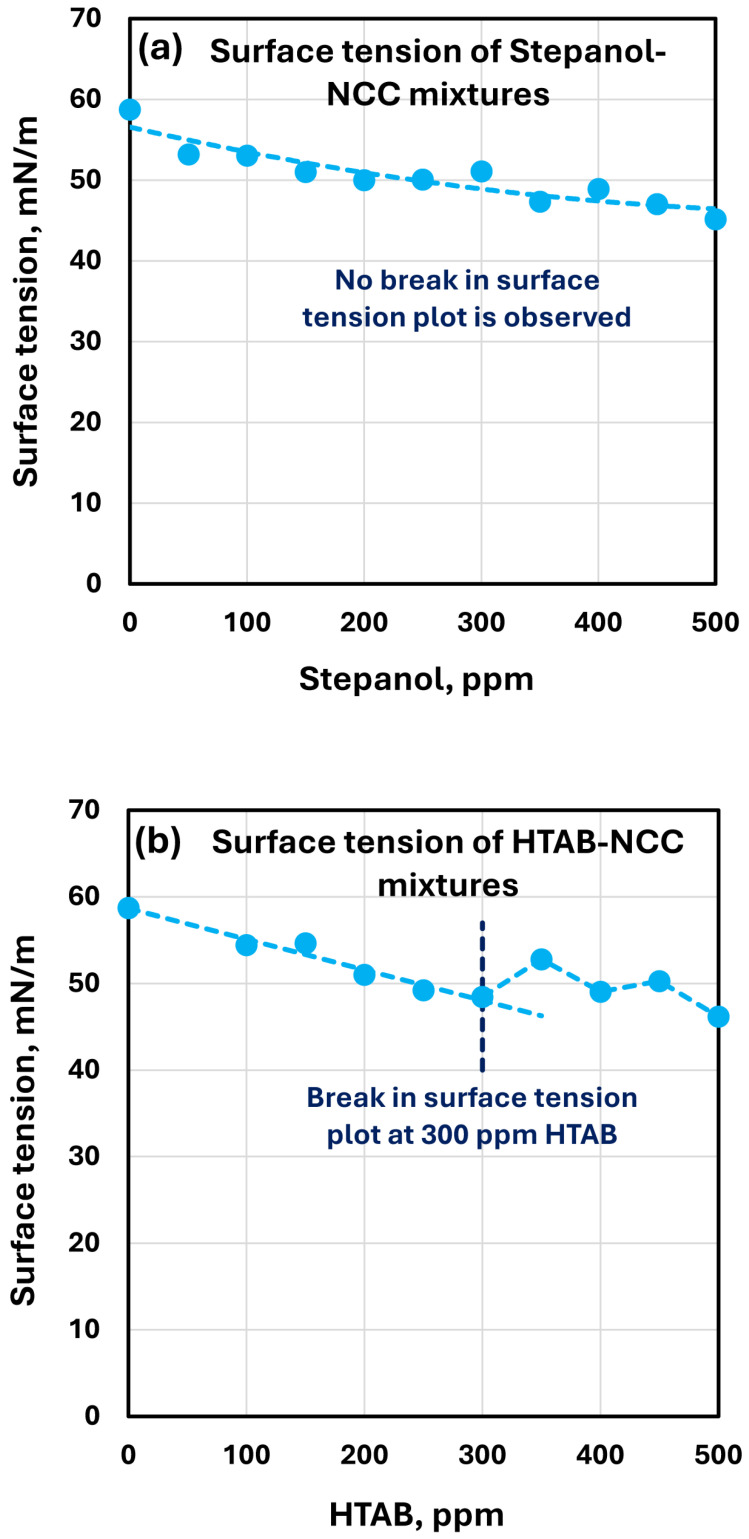
Surface tension variation in surfactant–NCC mixtures with the increase in surfactant concentration. (**a**) Surface tension of Stepanol–NCC mixtures; (**b**) surface tension of HTAB-NCC mixtures.

The surface tension plots of Stepanol–NCC and HTAB-NCC mixtures are shown in Figure 19. As expected, the surface tension decreases with the increase in surfactant concentration. While no break in the surface tension versus surfactant concentration plot is observed in the case of Stepanol–NCC mixtures, a clear break point is observed for the HTAB-NCC mixture around 300 ppm surfactant concentration. The surface tension rises somewhat at 300 ppm and tends to plateau. This is consistent with the rheology and conductivity measurements.

## 4. Suspensions of Fumed Silica Nanoparticles (N20)

### 4.1. Size Distribution of Fumed Silica Nanoparticles

**Figure 20:**
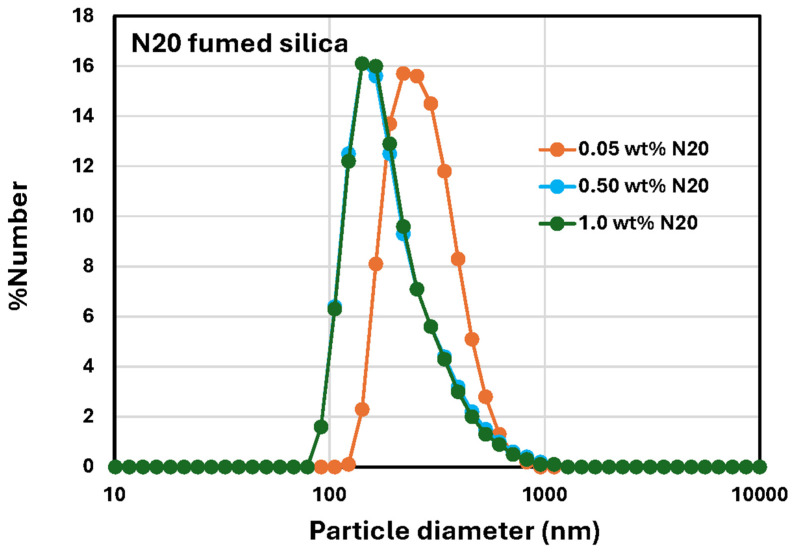
Size distribution of fumed silica nanosuspnsions at N20 concentrations of 0.05, 0.50, and 1.0 wt%.

Figure 20 shows the DLS data for fumed silica (N20) nanosuspensions at fumed silica concentrations of 0.05, 0.50, and 1.0 wt%. There is no clear trend with the variation in N20 concentration. The average hydrodynamic diameter of N20 is approximately 236 nm. The zeta potential of N20 is −37.3 mV.

### 4.2. Rheology of Suspensions of Fumed Silica (N20)

**Figure 21:**
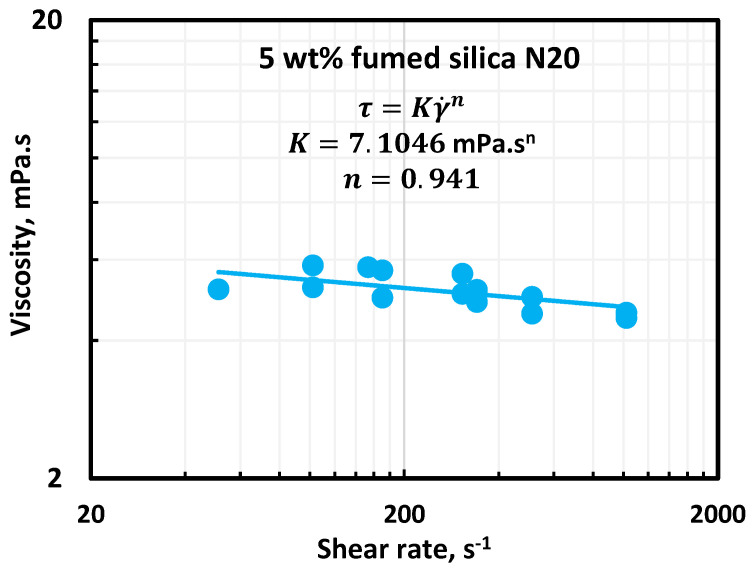
Rheological behavior of 5 wt% fumed silica N20 suspension.

**Figure 22:**
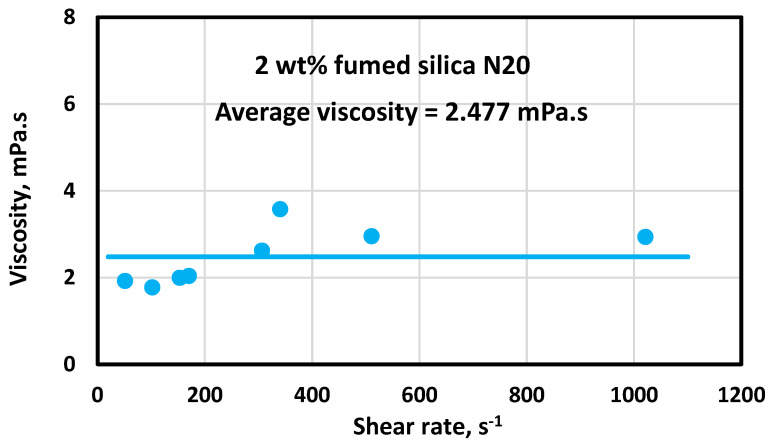
Viscosity versus shear rate data for 2 wt% fumed silica N20 suspension.

Figure 21 shows the rheological behavior of 5 wt% N20 suspension. The fumed silica suspension is slightly shear-thinning with power law constants as: n=0.941 and $K=7.1046\;mPa.s^{n}$. The 95% confidence intervals of n and K are $\left[0.9018,\;0.9807\right]$ and $\left[6.626,\;7.6186\right]$, respectively. The fumed silica suspension at 2 wt% N20 was Newtonian with a viscosity of 2.477 mPa.s, as shown in Figure 22. Upon comparison of Figure 7 and Figure 21, it is clear that the 1 wt% NCC suspension is much more viscous and shear-thinning as compared with the 5 wt% fumed silica N20 suspension. Although the exact reasons for this difference in rheological properties are not known, possible reasons are: (1) the cellulose nanocrystals (NCC) are rod-shaped particles whereas as fumed silica nanoparticles are more symmetric in shape, approximately spherical; and (2) nanocrystals are strongly aggregated whereas fumed silica nanoparticles are modestly aggregated. However, TEM/SEM images are required to support these claims about shape and aggregation of particles.

### 4.3. Influence of Surfactants on the Rheology of Fumed Silica N20 Suspensions

**Figure 23:**
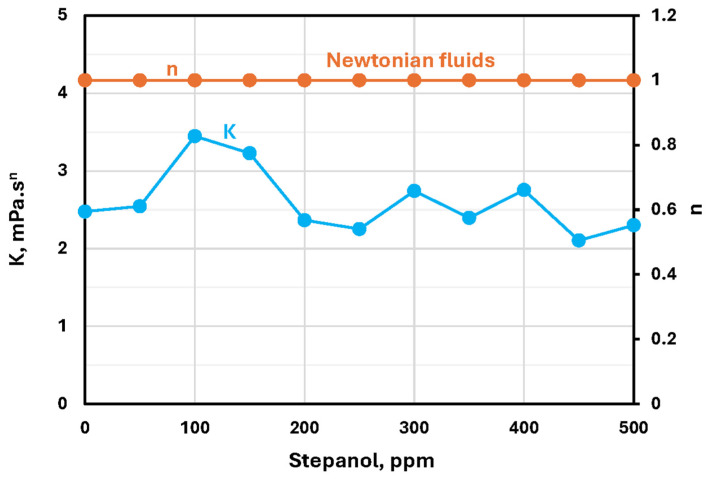
Variations in consistency and flow behavior indices of Stepanol-fumed silica (N20) mixtures with Stepanol concentration. The fumed silica (N20) concentration is fixed at 2 wt%.

**Figure 24:**
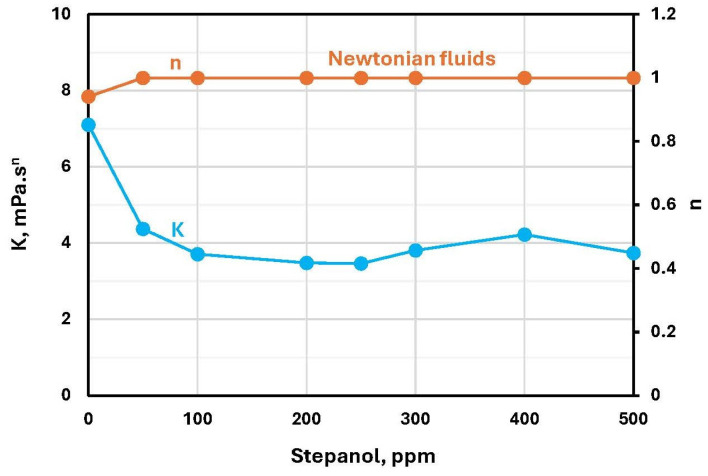
Variations in consistency and flow behavior indices of Stepanol-fumed silica (N20) mixtures with Stepanol concentration. The fumed silica (N20) concentration is fixed at 5 wt%.

Figure 23 and Figure 24 show the power law parameters (K and n) of Stepanol-fumed silica mixtures at 2 and 5 wt% N20. Note that these mixtures were mostly Newtonian as reflected in the value of n=1. Thus, the consistency index K in this case is simply the viscosity of the mixture. The consistency index, that is, viscosity in the present case, decreases with the increase in surfactant concentration to some extent especially at a high N20 concentration of 5 wt%.

**Figure 25:**
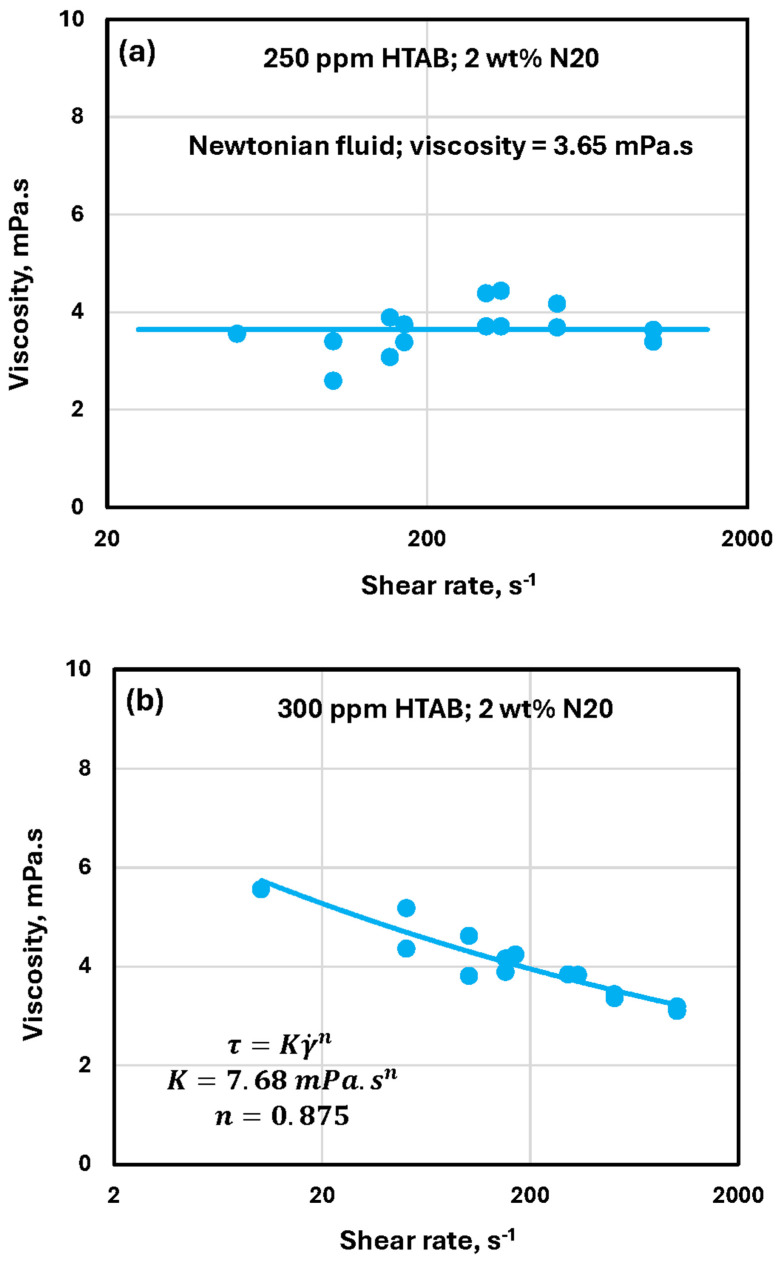
Rheological behavior of HTAB-fumed silica N20 mixtures at a fixed N20 concentration of 2 wt%. (**a**) 250 ppm HTAB. (**b**) 300 ppm HTAB.

Figure 25 shows the typical rheological behavior of HTAB-N20 mixtures at 2 wt% N20. The mixture is Newtonian up to a HTAB concentration of 250 ppm. With further increase in HTAB concentration, the mixtures become modestly shear-thinning.

**Figure 26:**
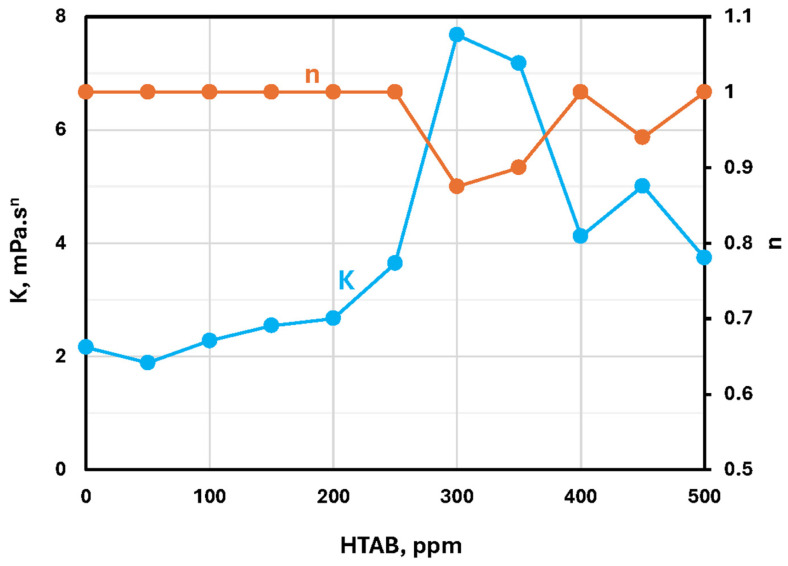
Variations in consistency and flow behavior indices of HTAB-fumed silica (N20) mixtures with HTAB concentration. The fumed silica (N20) concentration is fixed at 2 wt%.

Figure 26 shows the power law parameters (K and n) of HTAB-fumed silica mixtures at 2 wt% N20. The mixtures are Newtonian up to a HTAB concentration of 250 ppm. Above 250 ppm HTAB, the mixtures become shear-thinning to a modest extent. Most interestingly, the consistency index K rises sharply at 250 ppm and peaks at 300 ppm HTAB. With a further increase in HTAB concentration, the consistency index falls off. While the exact mechanisms for increase and fall in the consistency index in the range of 250 to 500 ppm HTAB are not known, possible mechanisms include: charge reversal (restabilization) of fumed silica nanoparticles, micelle formation competing with surface adsorption, and perhaps depletion flocculation.

**Figure 27:**
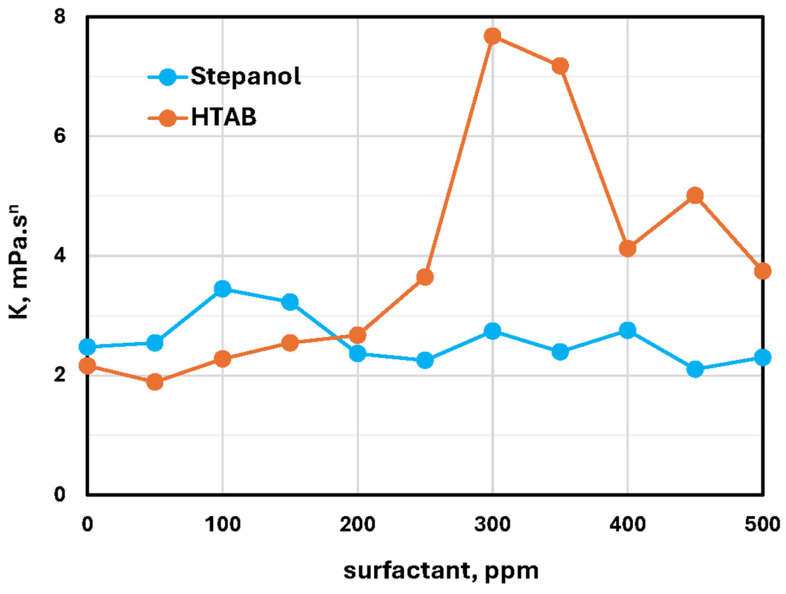
Comparison of consistency indices of Stepanol-fumed silica and HTAB-fumed silica mixtures. The fumed silica (N20) concentration is fixed at 2 wt%.

Figure 27 compares the consistency indices of Stepanol–N20 and HTAB-N20 mixtures. There is relatively negligible change in the consistency index of Stepanol–N20 mixtures with the increase in Stepanol concentration. The consistency index of HTAB-N20 mixtures is also nearly unchanged with the initial increase in surfactant concentration. However, starting at 250 ppm HTAB, the consistency index shoots up. After reaching a peak value at 300 ppm, the consistency index falls off with a further increase in HTAB concentration. The increase in the consistency index is indicative of structure formation due to interaction between oppositely charged fumed silica and surfactant HTAB. Similar behavior was observed in the case of HTAB-NCC mixtures (see Figure 12 and Figure 16). It appears that in the present case the structure is destroyed at high concentrations of HTAB.

**Figure 28:**
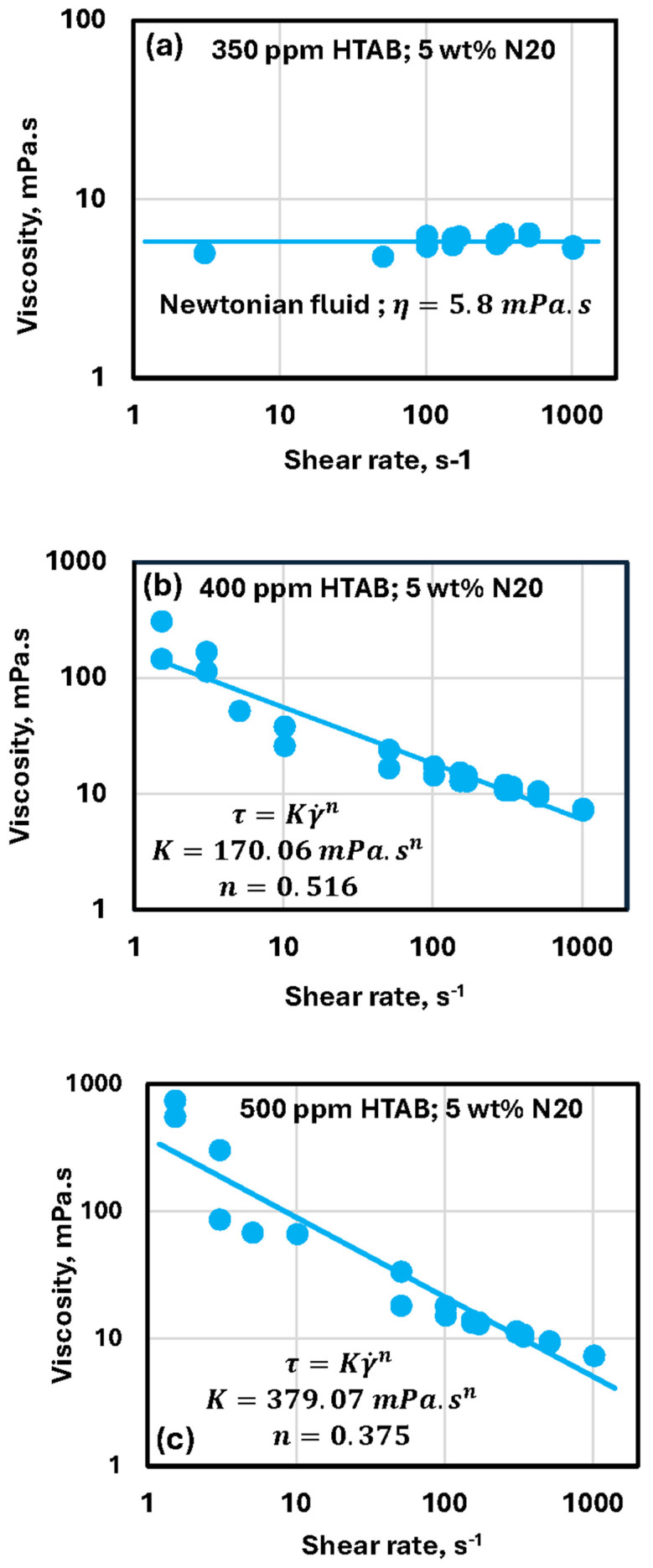
Rheological behavior of HTAB-fumed silica N20 mixtures at a fixed N20 concentration of 5 wt%. (**a**) 350 ppm HTAB. (**b**) 400 ppm HTAB. (**c**) 500 ppm HTAB.

Figure 28 shows the typical rheological behavior of HTAB-N20 mixtures at 5 wt% N20. The mixture is Newtonian up to a HTAB concentration of 350 ppm. With a further increase in HTAB concentration, the mixtures become highly shear-thinning.

**Figure 29:**
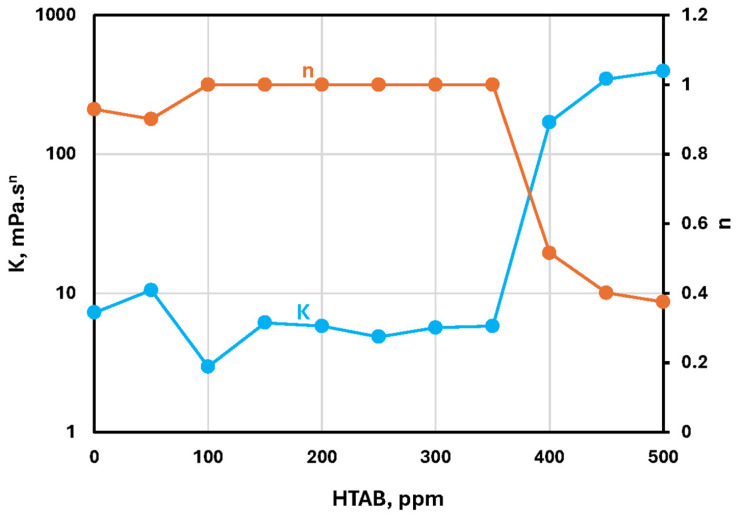
Variations in consistency and flow behavior indices of HTAB-fumed silica (N20) mixtures with HTAB concentration. The fumed silica (N20) concentration is fixed at 5 wt%.

Figure 29 shows the power law parameters (K and n) of HTAB-fumed silica mixtures at 5 wt% N20. The mixtures are Newtonian up to a HTAB concentration of 350 ppm. Above 350 ppm HTAB, the mixtures become highly shear-thinning. The consistency index K, however, rises sharply when the HTAB concentration exceeds 350 ppm. Above 450 ppm HTAB, the consistency index levels off.

**Figure 30:**
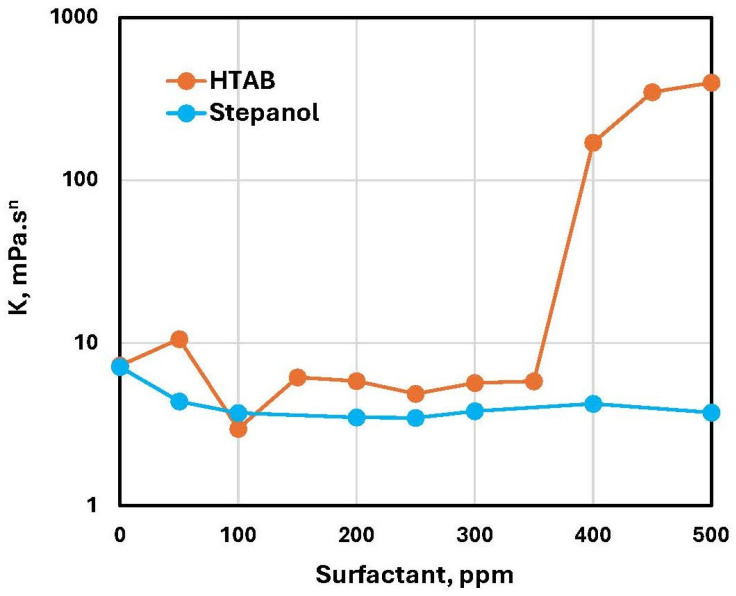
Comparison of consistency indices of Stepanol-fumed silica and HTAB-fumed silica mixtures. The fumed silica (N20) concentration is fixed at 5 wt%.

Figure 30 compares the consistency indices of Stepanol–N20 and HTAB-N20 mixtures. There is relatively negligible change in the consistency index of Stepanol–N20 mixtures with the increase in Stepanol concentration. The consistency index of HTAB-N20 mixtures is also nearly unchanged with the initial increase in surfactant concentration. However, starting at 350 ppm HTAB, the consistency index shoots up. After reaching a peak value at 450 ppm, the consistency index levels off with further increase in HTAB concentration. The increase in the consistency index is indicative of a three-dimensional network structure formation (similar to Figure 14) due to interaction between oppositely charged fumed silica and surfactant HTAB. Similar rheological behavior was exhibited by HTAB-NCC mixtures (see Figure 12).

### 4.4. Influence of Surfactants on the Electrical Conductivity and Surface Tension of Fumed Silica (N20) Suspensions

**Figure 31:**
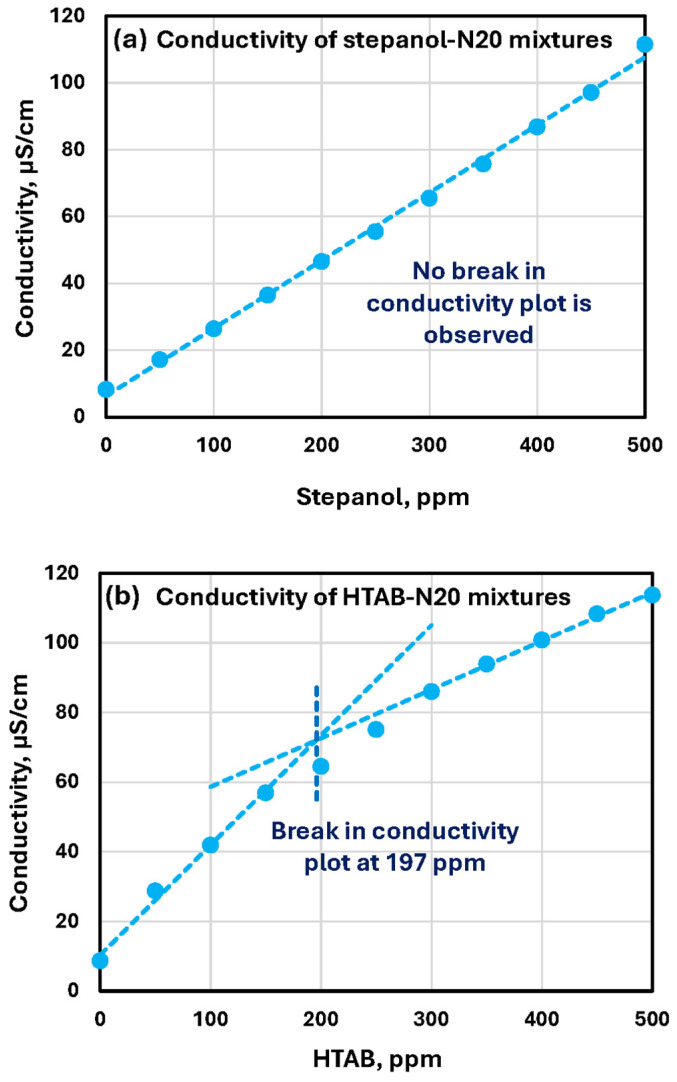
Electrical conductivity variation in surfactant–N20 mixtures with the increase in surfactant concentration. (**a**) Conductivity of Stepanol–N20 mixtures; (**b**) conductivity of HTAB-N20 mixtures. The fumed silica (N20) concentration is fixed at 2 wt%.

The electrical conductivity plots of Stepanol–N20 and HTAB-N20 mixtures are shown in Figure 31 at a 2 wt% fumed silica (N20) concentration. The conductivity of the Stepanol–N20 mixture increases linearly with the increase in Stepanol concentration without any break or change in slope (see Figure 31a). This is consistent with the variation in rheological properties of the Stepanol–N20 mixture with the increase in Stepanol concentration. No sharp changes were observed in rheological properties (see Figure 23). However, the conductivity plot of HTAB-N20 mixtures shows a different behavior (see Figure 31b). The conductivity of the HTAB-N20 mixture increases linearly up to about 200 ppm HTAB. At 197 ppm HTAB, a change in the slope of the conductivity plot occurs. Although the exact reason for the break point at 197 ppm HTAB is unknown, it could be due to the formation of micelles rather than adsorption on silica surfaces. At HTAB concentrations higher than 197 ppm, the conductivity increases slowly with the increase in HTAB concentration. This is consistent with the rheological data which exhibits a large change around a HTAB concentration of 200 ppm (see Figure 26). At HTAB concentrations larger than 200 ppm, the surfactant molecules either migrate to the surface of N20 nanoparticles resulting in charge neutralization or form micelles in the matrix fluid and hence we observe a slower increase in conductivity with the increase in HTAB concentration.

**Figure 32:**
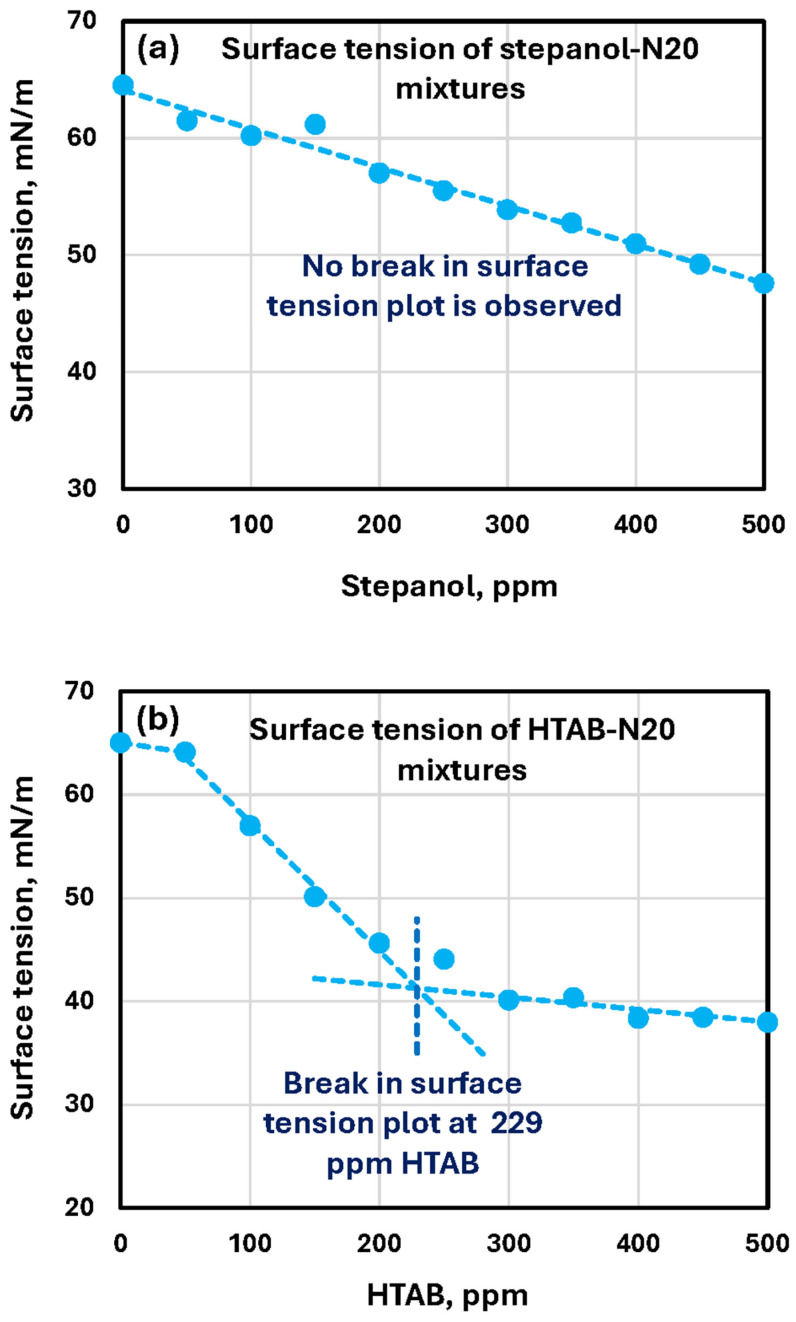
Surface tension variation in surfactant–N20 mixtures with the increase in surfactant concentration. (**a**) Surface tension of Stepanol–N20 mixtures; (**b**) surface tension of HTAB-N20 mixtures. The fumed silica (N20) concentration is fixed at 2 wt%.

The surface tension plots of Stepanol–N20 and HTAB-N20 mixtures are shown in Figure 32. As expected, the surface tension decreases with the increase in surfactant concentration. While no break in the surface tension versus surfactant concentration plot is observed in the case of Stepanol–N20 mixtures, a clear break point is observed for the HTAB-N20 mixture around 229 ppm surfactant concentration. This is consistent with the rheology and conductivity measurements (see Figure 27 and Figure 31).

**Figure 33:**
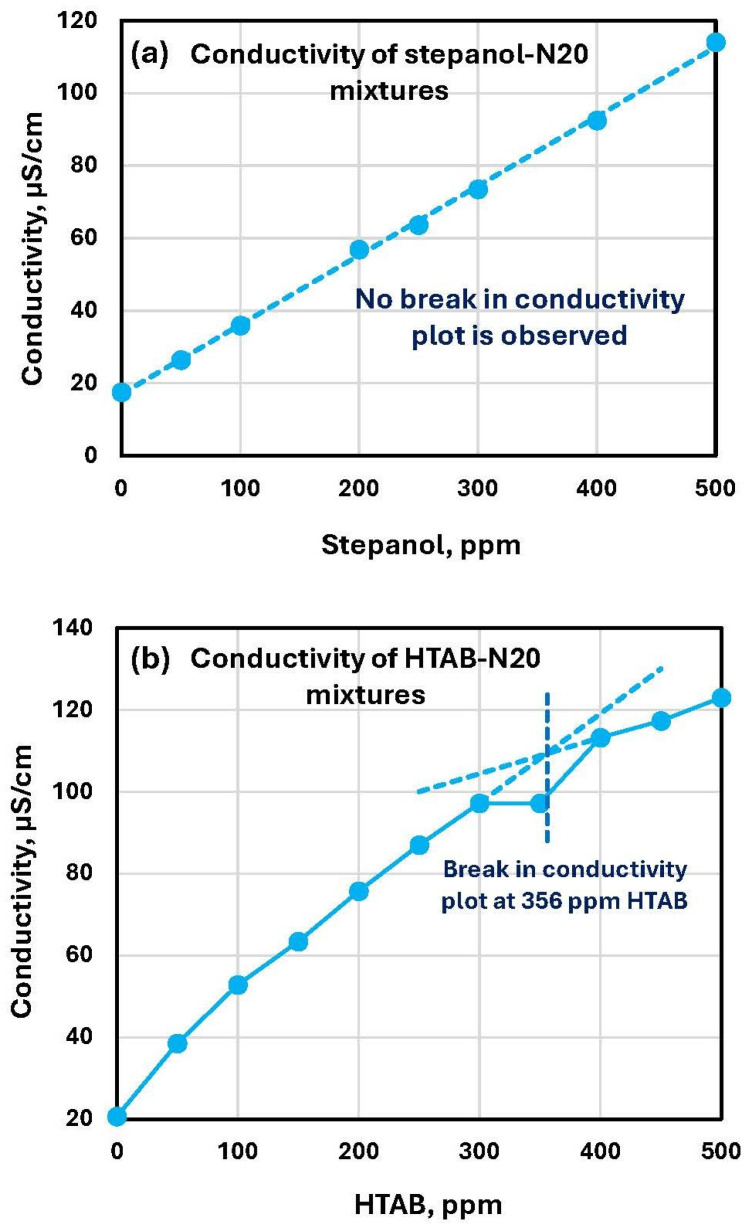
Electrical conductivity variation in surfactant–N20 mixtures with the increase in surfactant concentration. (**a**) Conductivity of Stepanol–N20 mixtures; (**b**) conductivity of HTAB-N20 mixtures. The fumed silica (N20) concentration is fixed at 5 wt%.

The electrical conductivity plots of Stepanol–N20 and HTAB-N20 mixtures are shown in Figure 33 at a 5 wt% fumed silica (N20) concentration. The conductivity of the Stepanol–N20 mixture increases linearly with the increase in Stepanol concentration without any break or change in slope (see Figure 33a). This is consistent with the variation in rheological properties of the Stepanol–N20 mixture with the increase in Stepanol concentration. No sharp changes were observed in rheological properties (see Figure 24). However, the conductivity plot of HTAB-N20 mixtures shows a different behavior (see Figure 33b). The conductivity of the HTAB-N20 mixture increases almost linearly up to about 300 ppm HTAB. At 356 ppm HTAB, a change in the slope of the conductivity plot occurs. At HTAB concentrations higher than 400 ppm, the conductivity increases slowly with the increase in HTAB concentration. This is consistent with the rheological data which exhibited a large change around a HTAB concentration of 350 ppm (see Figure 30). At HTAB concentrations larger than 400 ppm, the surfactant molecules migrate to the surface of N20 nanoparticles resulting in charge neutralization and hence we observe a slower increase in conductivity with the increase in HTAB concentration.

**Figure 34:**
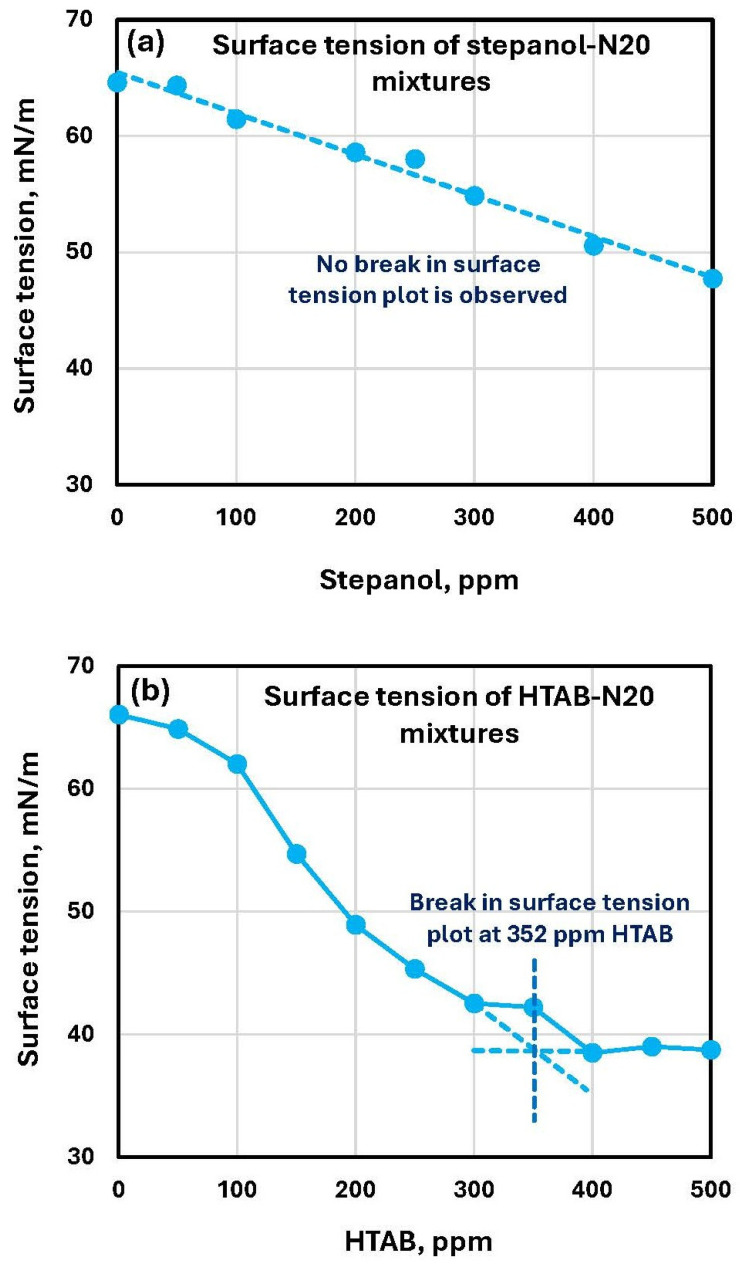
Surface tension variation in surfactant–N20 mixtures with the increase in surfactant concentration. (**a**) Surface tension of Stepanol–N20 mixtures; (**b**) surface tension of HTAB-N20 mixtures. The fumed silica (N20) concentration is fixed at 5 wt%.

The surface tension plots of Stepanol–N20 and HTAB-N20 mixtures are shown in Figure 34 at a 5 wt% fumed silica (N20) concentration. As expected, the surface tension decreases with the increase in surfactant concentration. While no break in the surface tension versus surfactant concentration plot is observed in the case of Stepanol–N20 mixtures, a clear break point is observed for the HTAB-N20 mixture around 352 ppm surfactant concentration. This is consistent with rheology and conductivity measurements (see Figure 30 and Figure 33).

## 5. Conclusions

The interactions between two oppositely charged surfactants (anionic and cationic) and suspensions of cellulose nanocrystals and fumed silica nanoparticles were investigated experimentally. The unique feature of this work is the simultaneous measurement of the rheology, surface activity, and electrical conductivity of nanosuspensions to draw conclusions about the interactions. Furthermore, the measurements were restricted to low surfactant concentrations in the range of 0 to 500 ppm. Based on the experimental results, the conclusions of this study are summarized as follows:

- Weak interactions occur between NCC and Stepanol. No clear trend in the variation in rheological properties with surfactant concentration. No breaks in conductivity-versus-surfactant-concentration and surface-tension-versus-surfactant-concentration plots. The interactions are weak due to the same charge (negative) on the NCC and surfactant.
- Strong interactions occur between NCC and HTAB when surfactant concentration exceeds 300 ppm. Sharp changes in the rheological properties occur. Clear break points in conductivity-versus-surfactant-concentration and surface-tension-versus-surfactant-concentration plots observed around a surfactant concentration of 300–350 ppm. The interactions are strong due to an opposite charge on the NCC (negative) and surfactant (positive). Due to charge neutralization, a three-dimensional network structure of cellulose nanocrystals is formed. The HTAB-NCC mixtures also possess a yield stress at high HTAB concentrations (HTAB ≥400 ppm).
- Weak interactions between fumed silica N20 and Stepanol. No sharp changes in the variation in rheological properties with surfactant concentration. No breaks in conductivity-versus-surfactant-concentration and surface-tension-versus-surfactant-concentration plots. The interactions are weak due to the same charge (negative) on the fumed silica N20 and surfactant.
- Strong interaction between fumed silica N20 and HTAB when surfactant concentration exceeds 200–250 ppm at a fixed N20 concentration of 2 wt%. Sharp changes in rheological properties occur when surfactant concentration exceeds 200–250 ppm. Clear break points in conductivity-versus-surfactant-concentration and surface-tension-versus-surfactant-concentration plots observed around a surfactant concentration of 197–229 ppm. At a higher fumed silica N20 concentration of 5 wt%, a strong interaction between fumed silica N20 and HTAB occurs when surfactant concentration exceeds 350 ppm. There are sharp changes in rheological properties when surfactant concentration exceeds 350 ppm. Break points in conductivity versus surfactant concentration and surface tension versus surfactant concentration plots observed around a surfactant concentration of 352–356 ppm. The interactions are strong due to an opposite charge on the fumed silica N20 (negative) and surfactant (positive). Changes in the rheological properties and the break points in conductivity and surface tension plots are due to the structure formation in the system.
- The break points observed in 2 wt% N20 and 1 wt% NCC upon addition of HTAB occur at different HTAB concentrations, about 229 ppm for fumed silica and 300 ppm for NCC, are due to differences in surface area, charge density, and hydrophobicity of fumed silica and NCC.
- The results of this work offer valuable insights into tailoring surfactant–nanoparticle systems for industrial applications, where precise control of rheological and interfacial properties is essential. The interplay between nanoparticle charge and type, surfactant charge and type, and concentrations plays a critical role in optimizing surfactant–nanoparticle systems for industrial applications. Strong electrostatically attractive interactions between oppositely charged cationic surfactants and negatively charged nanocrystals and nanoparticles greatly enhance rheological properties.
- Future research in this area should focus on elucidating the mechanisms behind the changes observed in rheological and interfacial properties. The gelling behavior of the HTAB-NCC system observed at high surfactant concentrations should be investigated further using dynamic (oscillatory) rheological measurements. Storage and loss moduli measured using frequency sweep would provide useful insights about the microstructure. Stress sweep and creep/recovery experiments would also be equally important in probing the microstructure. For example, stress sweep can provide direct information about the yield stress of the fluid. Regarding the shape and size of individual nanoparticles and aggregates of nanoparticles, TEM/SEM imaging and SAXS would be very useful, both before and after structural changes. An accurate measurement of Zeta potential is equally important, both before and after structural changes. The adsorption behavior of surfactants on fumed silica and NCC will provide additional insights into the mechanisms.

## Figures and Tables

**Table 1:** Compositions of suspensions of nanocrystalline cellulose (NCC) and fumed silica (N20) investigated in this study.

Nanoparticle Type	Nanoparticle Concentration (wt%)	Surfactant Type	Surfactant Concentration (ppm)
Nanocrystalline cellulose (NCC)	1.0	Stepanol	Eleven concentrations: 0, 50, 100, 150, 200, 250, 300, 350, 400, 450, 500
Nanocrystalline cellulose (NCC)	1.0	HTAB	Eleven concentrations: 0, 50, 100, 150, 200, 250, 300, 350, 400, 450, 500
Fumed silica (N20)	2.0	Stepanol	Eleven concentrations: 0, 50, 100, 150, 200, 250, 300, 350, 400, 450, 500
Fumed silica (N20)	5.0	HTAB	Eleven concentrations: 0, 50, 100, 150, 200, 250, 300, 350, 400, 450, 500

**Table 2:** Power law parameters for Stepanol–NCC mixtures.

Stepanol, ppm	K, mPa.s^n^	n	$R^{2}$ (Correlation Coefficient)
0	96.336	0.488	0.9884
50	30.432	0.661	0.758
100	43.342	0.542	0.8754
150	28.9	0.625	0.8607
200	77.229	0.439	0.91
250	123.86	0.419	0.98
300	112.6	0.433	0.857
350	69.476	0.494	0.9684
400	63.2	0.545	0.9441
450	82.609	0.474	0.9547
500	92.397	0.462	0.9137

**Table 3:** Power law parameters for HTAB-NCC mixtures.

HTAB, ppm	K, mPa.s^n^	n	$R^{2}$ (Correlation Coefficient)
0	96.336	0.488	0.9884
50	93.029	0.521	0.9951
100	78.203	0.527	0.9651
150	47.415	0.599	0.7904
200	105.75	0.58	0.8885
250	77.823	0.595	0.9102
300	302.86	0.449	0.986
350	1053.8	0.288	0.9964
400	1740	0.227	0.9906
450	1790.8	0.235	0.9962
500	1407.7	0.279	0.9912

## Data Availability

The original contributions presented in this study are included in the article. Further inquiries can be directed to the corresponding author.
